# Lead-exposure associated miRNAs in humans and Alzheimer’s disease as potential biomarkers of the disease and disease processes

**DOI:** 10.1038/s41598-022-20305-5

**Published:** 2022-09-24

**Authors:** Qingfeng Wen, Marcha Verheijen, Mandy Melissa Jane Wittens, Julia Czuryło, Sebastiaan Engelborghs, Duncan Hauser, Marcel H. M. van Herwijnen, Thomas Lundh, Ingvar A. Bergdahl, Soterios A. Kyrtopoulos, Theo M. de Kok, Hubert J. M. Smeets, Jacco Jan Briedé, Julian Krauskopf

**Affiliations:** 1 Department of Toxicogenomics, Maastricht University, Universiteitssingel 50, 6229 ER Maastricht, The Netherlands; 2 MHeNS, School for Mental Health and Neuroscience, Maastricht University, Universiteitssingel 50, 6229 ER Maastricht, The Netherlands; 3 Department of Biomedical Sciences, Institute Born-Bunge, University of Antwerp, Universiteitsplein 1, 2610 Antwerpen, Belgium; 4 Neuroprotection and Neuromodulation (NEUR), Center for Neurosciences (C4N), Vrije Universiteit Brussel (VUB), Laarbeeklaan 103, 1090 Brussel, Belgium; 5 Department of Neurology, and Brussels Integrated Center for Brain and Memory (Bru-BRAIN), Universitair Ziekenhuis Brussel (UZ Brussel), Laarbeeklaan 101, 1090 Brussel, Belgium; 6 Division of Occupational and Environmental Medicine, Lund University Hospital, Lund, Sweden; 7 Section of Sustainable Health, Department of Public Health and Clinical Medicine, Umeå University, Umeå, Sweden; 8 Institute of Chemical Biology, National Hellenic Research Foundation, Athens, Greece; 9 School for Oncology and Developmental Biology (GROW), Maastricht University, Maastricht, The Netherlands

**Keywords:** Alzheimer's disease, Environmental impact, Gene expression, Computational biology and bioinformatics, Biomarkers

## Abstract

Alzheimer’s disease (AD) is a neurodegenerative disease that eventually affects memory and behavior. The identification of biomarkers based on risk factors for AD provides insight into the disease since the exact cause of AD remains unknown. Several studies have proposed microRNAs (miRNAs) in blood as potential biomarkers for AD. Exposure to heavy metals is a potential risk factor for onset and development of AD. Blood cells of subjects that are exposed to lead detected in the circulatory system, potentially reflect molecular responses to this exposure that are similar to the response of neurons. In this study we analyzed blood cell-derived miRNAs derived from a general population as proxies of potentially AD-related mechanisms triggered by lead exposure. Subsequently, we analyzed these mechanisms in the brain tissue of AD subjects and controls. A total of four miRNAs were identified as lead exposure-associated with hsa-miR-3651, hsa-miR-150-5p and hsa-miR-664b-3p being negatively and hsa-miR-627 positively associated. In human brain derived from AD and AD control subjects all four miRNAs were detected. Moreover, two miRNAs (miR-3651, miR-664b-3p) showed significant differential expression in AD brains versus controls, in accordance with the change direction of lead exposure. The miRNAs’ gene targets were validated for expression in the human brain and were found enriched in AD-relevant pathways such as axon guidance. Moreover, we identified several AD relevant transcription factors such as CREB1 associated with the identified miRNAs. These findings suggest that the identified miRNAs are involved in the development of AD and might be useful in the development of new, less invasive biomarkers for monitoring of novel therapies or of processes involved in AD development.

## Introduction

Alzheimer’s disease (AD) is the most common and rapidly growing form of dementia estimated to reach 152 million cases by year 2050^1^. It is a progressive neurodegenerative disease characterized by a gradual memory loss over time. The first stage of AD is the preclinical stage, which can last from ten to twenty years in the absence of clinical symptoms^2^; next is the prodromal stage, which occurs with evident cognitive decline. Lastly the dementia stage starts, with progressive decline in thinking, memory and cognitive skills, due to overall neurodegeneration^3^. The pathology of AD is characterized by the accumulation of Amyloid β (Aβ) aggregates that form plaques in the brain, and the hyperphosphorylation of the tau protein leading to intraneuronal neurofibrillary tangles (NFTs), eventually resulting in the death of neurons^4^. AD can be familial with onset of the disease at an early age due to autosomal dominant gene mutations that cause AD. It can also be sporadic with a later onset, mostly caused by aging in combination with genetic and environmental risk factors which may include pesticides^5^, air pollutants^6^, viruses^7^, and the heavy metals lead^8^ and cadmium^9^.

Currently, the diagnostic tools of AD include magnetic resonance imaging (MRI), positron emission tomography (PET) imaging, and cerebrospinal fluid (CSF) biomarkers (Aβ1-42, Aβ1-42/Aβ1-40 ratio, T-tau and P-tau levels)^10^. However, except for CSF biomarkers, these tools are related to a relatively later stage in the progression of AD. When AD is clinically diagnosed, neuronal loss has already occurred in many brain regions^11^. Therefore, developing early biomarkers in blood that are less invasive is very important, as that might allow extensive screening in populations for individuals at risk of AD and facilitate the development of potential early intervention strategies.

A growing body of literature suggests that exposure to heavy metals may influence the onset and progression of AD^8,9,12,13^. Lead and cadmium have the potential to disturb the homeostasis of many pathways in human cells. Exposure to lead can originate from many sources, for example by paint, gasoline, soil, or dust^14^, whereas exposure to cadmium can occur due to combustion of fossil fuels, phosphate fertilizers, diet, or smoking^8^. Exposure to these heavy metals can alter neuronal function, dysregulate genes, damage mitochondria, and cause neuroinflammation, cognitive impairment and Aβ accumulation^8,14^. Additionally, lead exposure can have an impact on several cellular pathways, such as phosphatidylinositol 3 kinase-protein kinase B (PI3K-Akt) pathway that is linked to AD development^15,16^ since Aβ oligomers inhibit the PI3K-Akt pathway, and inhibition of the PI3K/AKT pathway could cause neuron death and eventually dementia. Moreover, a lead effect may operate many decades before the clinical disease appears, since prenatal lead exposure of primate fetuses can cause epigenetic changes in the expression of Aβ and tau^17^, that is also supported by the observations of Aβ in adults who had been exposed prenatally^18^.

MiRNAs that are small non-coding RNAs of about 20 nucleotides in length have emerged as a new class of biomarkers. They are abundantly present in all tissues, cell types, and even biofluids and exert their function in posttranscriptional regulation of gene expression by repressing mRNA translation^19^. MiRNAs are involved in crucial processes in many diseases, including central nervous system (CNS) disorders^20^. They have numerous functions in the human brain including dendritic branching, receptor regulation and spine maturation^21,22^. In addition, miRNAs are linked to AD by directly affecting the underlying pathogenic pathways, for example by targeting APP^23^ or BACE1 expression^24,25^. Therefore, detection of specific miRNAs can be used as potential biomarkers for CNS diseases^26–28^.

Several studies have examined whether certain miRNA could serve as biomarkers for AD^29,30^. For example, miR-29 is involved in neuronal survival, maturation, proliferation, and plasticity; another example is miR-34a-5p that is responsible for cell survival, apoptosis, neuroprotection, and signalling; yet another, miR-132-3p, is also involved in neuronal plasticity^20^. MiRNAs are also involved in regulating blood–brain barrier permeability^31^. Besides, some miRNAs have shown significantly differential expression in AD versus controls in different specimen types, such as brain and blood^32^, and these differentially expressed miRNAs could be potential biomarkers^33–35^. However, results of these studies were inconsistent with a lack of reproducibility and validation of candidate miRNAs across studies^29,36^. Here, we applied a new workflow to discover miRNA biomarkers, triggered by known risk factors for AD such as exposure to heavy metals^37–39^. Heavy metal exposure has previously been shown to affect miRNAs expression^40^. By examining which miRNAs are affected by heavy metal exposure, and the associations between these miRNAs and AD, also investigating the corresponding pathways they are involved in, we might be able to identify molecular mechanisms involved in AD development. As these metals also affect neurodevelopment in children and fetuses, these mechanisms might also be of interest for the understanding of these early effects.

**Figure 1:**
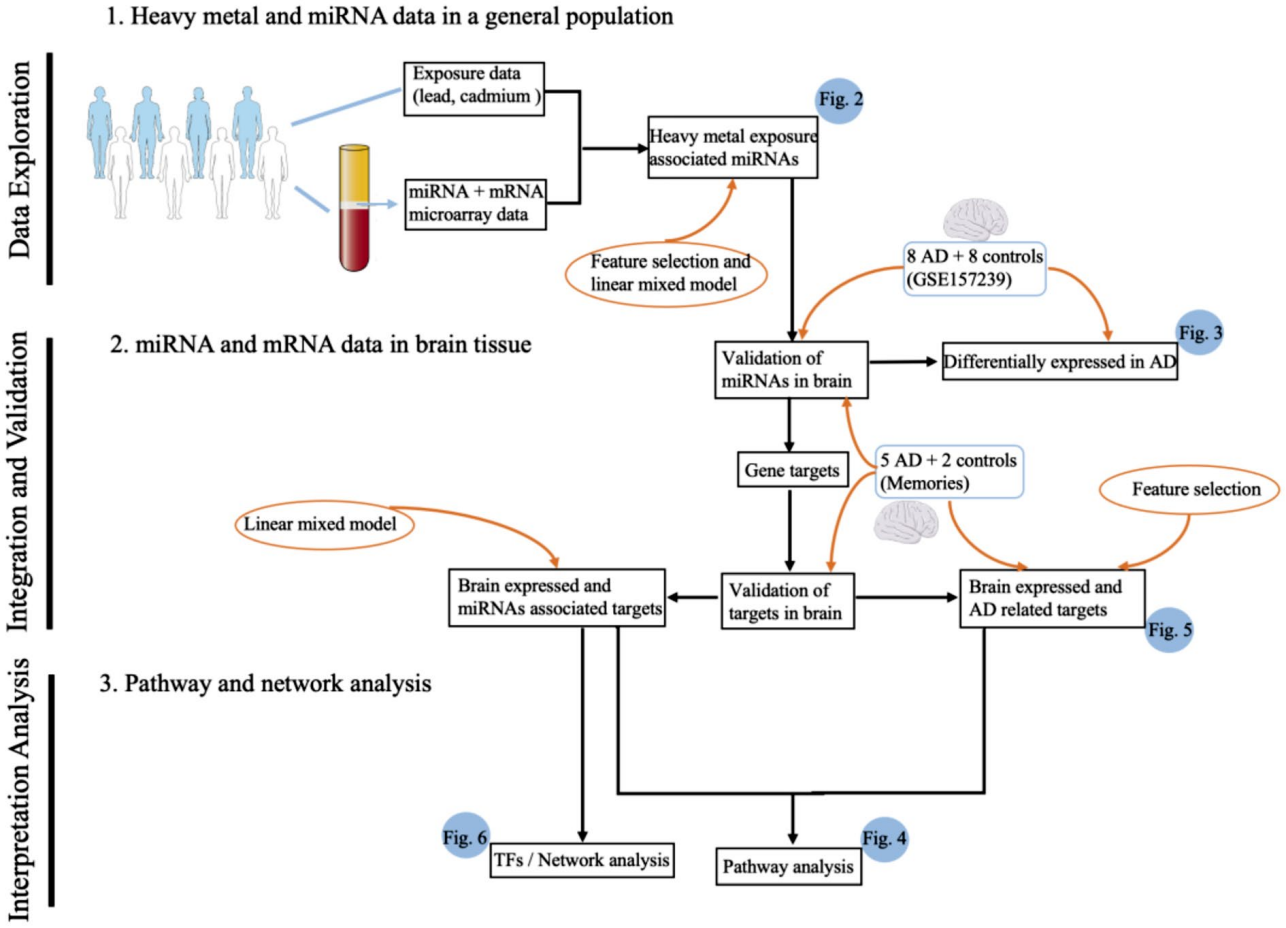
Workflow of the study. (1) We explored heavy metal (lead and cadmium) associated miRNAs in blood from a general population derived from the NSHDS by using feature selection and linear mixed model. (2) The resulting miRNAs were validated for expression in human brain by data from Memories, and publicly available dataset with larger sample size for different brain regions was used as additional validation and checked for differential expression. Subsequently, we derived experimentally validated gene targets for these miRNAs, which were also validated for expression in human brain (data derived from the Memories project). Next, we selected gene targets that showed an association with the identified miRNAs in transcriptomics data of NSHDS by using linear mixed model, and selected AD related gene targets based on the transcriptomics data of Memories project by using feature selection. (3) The identified gene targets were subjected to pathway analysis to elucidate molecular mechanisms of heavy metal-associated miRNAs. Moreover, we performed network analysis to interpret complex miRNAs-TFs-targets regulation.

The aim of this study is to investigate whether exposure to lead and cadmium is associated with specific miRNA perturbations, and subsequently if a deregulation of these miRNAs is also observed in AD patients. Our assumption is that molecular response upon heavy metal exposure of blood cells on the miRNAs level may reflect the response of neurons. The study design is shown in Fig. 1. First, we screened for miRNAs associated with lead and cadmium exposure in a human population study of 186 subjects from the Northern Sweden Health and Disease Study (NSHDS). The miRNAs identified were then analyzed in brains of AD patients and controls. Lastly, we used the integration of blood-derived miRNA and mRNA in combination with brain-derived mRNA data from the Memories project to identify potential mechanisms triggered by lead in the brain. For this analysis, we used data from two different EU projects (the FP7 EnviroGenomarkers project and the Interreg Memories project) and a publicly available dataset^41^.

## Materials and methods

### The data sources

The population data was retrieved from NSHDS^42^, in which its subcohort the Vasterbotten Intervention Programme^43^ was used. NSHDS is one of the cohorts included in the European FP7 EnviroGenomarkers project^44^. In this project, blood samples, exposure data (including lead, cadmium, and organic pollutants) and health data (age, BMI, gender, education, smoking, alcohol, and activity) were collected when subjects were healthy. In total, 226 buffy coat samples were used for miRNA and mRNA data analysis. A detailed description of the study population can be found in a previous publication^45^. The concentrations of lead and cadmium were measured with inductively coupled plasma-mass spectrometry (ICP-MS; Thermo X7, Thermo Elemental, Winsford, UK) in erythrocyte samples with 0.123 ~ 5.224 µg/L for cadmium and 14.209 ~ 149.193 µg/L for lead^46^. It should be noted that the corresponding concentration in original whole blood is roughly half of the concentration in erythrocytes, as both cadmium and lead mainly resides in the erythrocytes that make up almost half of the blood volume. Total RNA was extracted from buffy coats, and the analysis and processing of both miRNA (Agilent 8 × 60 K human miRNA microarray) and mRNA data (Agilent 4 × 44 K human whole genome microarray) were all mentioned in an earlier study^45^. After removing outliers and missing values in exposure/health data, 186 subjects were left for the analysis. To calculate the statistical power of the sample size, we performed power calculation using R package “ssize.fdr”^47^. With the power of 80% at the 10% significance level, 160 subjects were needed, which means 186 samples in our study should be enough to achieve the required statistical power. In total, expression data for 543 miRNAs and 15,804 genes were provided. The EnviroGenomarkers project and its associated studies and protocols were approved by the Regional Ethical Review Board of the Umea Division of Medical Research and all participants gave written informed consent. All methods were performed in accordance with the relevant guidelines and regulations.

From the EU Interreg Memories project^48^, the miRNA and mRNA sequencing data were available^49^, including samples from three brain regions [Gyrus cinguli (Gc), Gyrus temporalis superior (Gt) and Brodmann a4 or a6 (Br)] of five AD cases and two controls. The clinical information of these seven participants is shown in Table 1, among which onset age refers to the age at first symptoms, death age refers the age when the patient died, and disease duration is the time frame between these two timepoints. Publicly available dataset GSE157239^41^ was used as additional expression validation of miRNAs in human brains due to the larger sample size (eight AD vs eight Controls), the detailed information is presented in Supplementary materials (Table S1).

**Table 1:** The information of AD cases and controls in validation dataset.

Case	Onset age	Death age	Duration (years)	Braak stage
AD1	69	72	3	III
AD2	70	72	2	V
AD3	67	71	4	IV
AD4	80	84	4	V–IV
AD5	77	78	1	III
NonAD1	–	78	–	I
NonAD2	–	80	–	0(Aβ), I(Tau)

### Analytical procedures and statistics

The analytical workflow of this study is shown in Fig. 1. All analyses were conducted by the open-source software R (version 4.1.0) and Bioconducter^50^. Many covariates such as age and gender have an impact on miRNA expression and some covariates are related to each other. Therefore, we considered both confounders and interactions in linear mixed model to define the impact of heavy metals exposure on miRNA expression based on data from the EnviroGenomarkers project. Batch effect of microarray data was also considered.

Limma package on R was used to recognize each covariate’s effect on miRNA expression. Then, Quantile–Quantile (QQ) plots as shown in Supplementary Fig. S1 were utilized to interpret the results. From the QQ plots, we could recognize which p-values do not conform to normal distribution that means the covariate should be considered as a confounder. As a result, BMI, age, smoking, gender, alcohol, education, zScore and activity were considered as confounders. Thereinto, zScore was the representative of persistent organic pollutants including all measured co-exposures comprising polychlorinated biphenyls (PCBs), hexachlorobenzene (HCB) and DDE (a common dichlorodiphenyltrichloroethane metabolite)^45^. However, education and cadmium data did not conform to the normal distribution, and no miRNAs were affected by these two covariates (Supplementary Table S2). Thus, we excluded education and cadmium from the model and only considered lead exposure in the following analysis. Based on Pearson Correlation Coefficients, age and BMI, age and zScore, as well as zScore and lead were correlated to each other separately, so these pairs were considered as interactions^51^ (Supplementary Fig. S3).

The glmnet (version 4.1-1) package in R was used to operate feature selection based on elastic net regression algorithm to filter lead exposure relevant miRNAs^52^. Then, we used a linear mixed model provided by the R package lme4 (version 1.1-27) to determine miRNAs significantly associated with the exposure intensity^53^. MiRNAs with a false discovery rate (FDR) below 0.1 were considered to be significant. The expression of lead associated miRNAs in human brain was validated via GSE157239, additionally, differential expression analysis was conducted to obtain differentially expressed miRNAs in AD versus controls based on R package limma (version 3.48.0)^54^. MiRNAs with a p-value below 0.05 were considered as significantly differentially expressed miRNAs.

From the database miRTarBase (release 8.0), we retrieved all gene targets of miRNAs which were lead exposure associated^55^. The associations between miRNAs and their targets were obtained via linear mixed model based on miRNA and mRNA microarray data from the EnviroGenomarkers project, and these genes were considered as lead associated. The expression of lead associated miRNAs’ target genes in human brain was validated via mRNA sequencing data from the Memories project. Feature selection as described above was performed to filter AD relevant genes. Lastly, the R package pheatmap (version 1.0.12) was used to visualize gene expression in human brains. Figure 1 as partly generated using Servier Medical Art, provided by Servier, licensed under a Creative Commons Attribution 3.0 Unported License (https://creativecommons.org/licenses/by/3.0/).

### Data integration and network analysis

We specifically focused on transcription factors (TFs) via a network analysis, because these genes play a major role in the regulation of gene expression^56^. For TFs-miRNAs regulations and TFs-targets regulations, we utilized TransmiR v2^57^ and TRRUST v2 databases^58^, separately. The miRNAs’ 5p or 3p information was not considered since TransmiR v2 database did not have this kind of information. All these interactions were exported to Cytoscape (version 3.8.2) to be visualized^59^. Pathway analysis was performed by over-representation analysis in ConsensusPathDB (release 35)^60^, only focusing on KEGG database. Pathways with a q.value below 0.1 were considered to be significant^61^. The display of these pathway were based on ggplot2 in R^62^.

## Results

We examined the impact of lead exposure on miRNA expression from 186 buffy coat samples of healthy subjects. To filter important lead exposure related miRNAs, we conducted feature selection based on elastic net regression algorithm^63^ (Supplementary Fig. S2). In total 26 miRNAs were selected. Subsequently, a linear mixed model was used to identify the relationship between lead exposure and miRNA expression. Eventually, four miRNAs were significantly associated (FDR < 0.1) to lead exposure, with hsa-miR-3651, hsa-miR-150-5p and hsa-miR-664b-3p being negatively and hsa-miR-627 positively associated to lead levels. As mentioned earlier, cadmium was excluded in the following analysis because it did not follow a normal distribution and no miRNAs were associated to cadmium levels.

**Figure 2:**
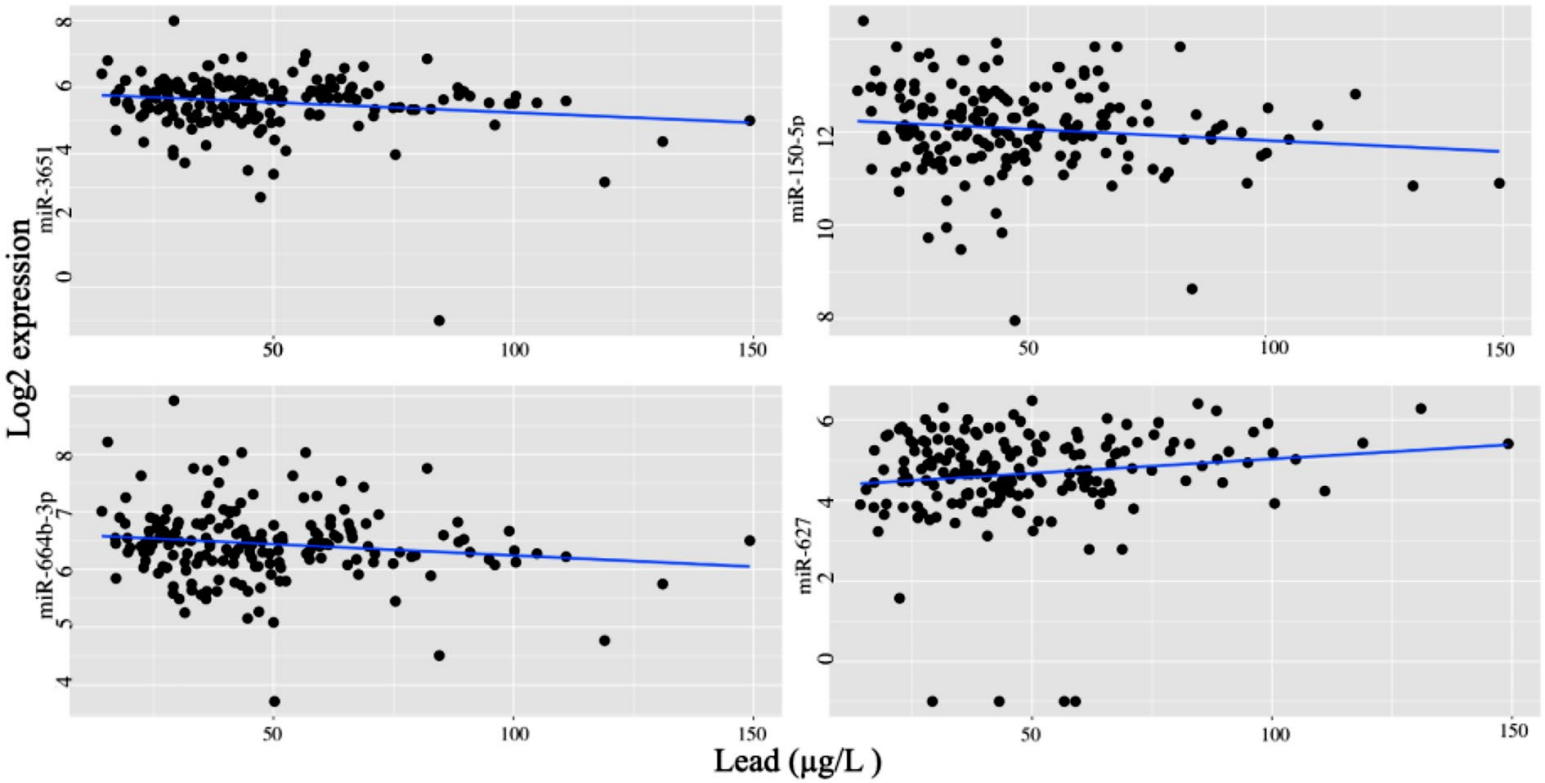
The scatterplot showed expression levels of four miRNAs associated with lead exposure. Scatter plot of the expression of 4 miRNAs (miR-3651, miR-664b-3p, miR-150-5p, and miR-627), identified in the EnviroGenomarker dataset as associated with the lead concentration via a linear mixed model, and plotted against the lead concentration in whole blood.

In order to visualize the effect of lead exposure on the miRNA expression, we used the scatterplot to show the association of these four miRNAs and lead exposure (Fig. 2). Even we did not exhibit the effect of confounders like age and gender, we can still find the small but clear trend of the association between these miRNAs and lead exposure.

### Lead exposure associated miRNAs expressed in human brains

**Figure 3:**
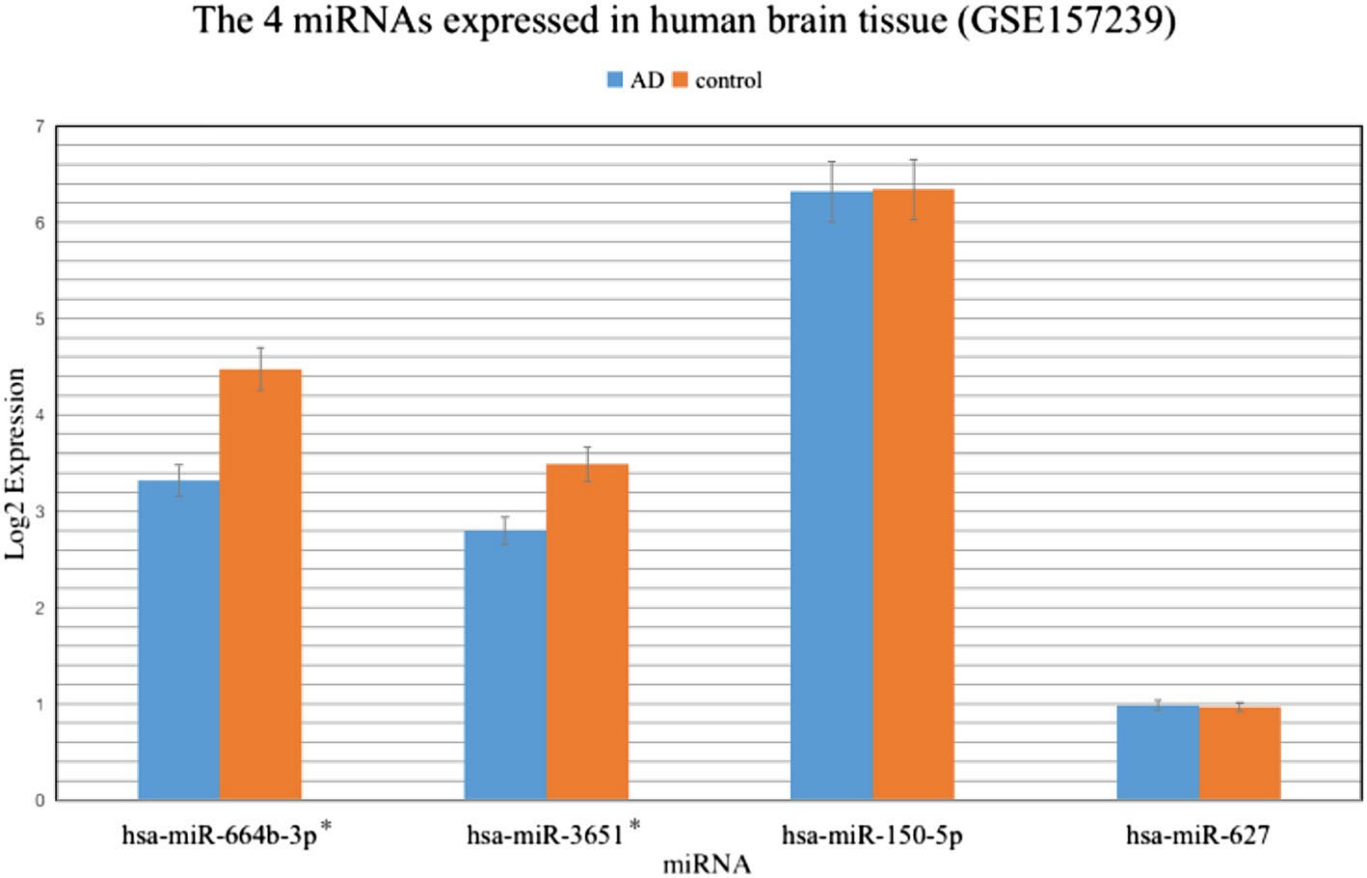
The expression of lead exposure associated miRNAs in human brain tissue from the miRNA microarray dataset GSE157239. Two miRNAs (miR-664b-3p and miR-3651) were significantly differentially expressed in brains of AD comparing with controls. All show expression in the same direction in AD cases compared to lead exposed cases. *Means significantly differential expression with p-value < 0.05.

We assumed that molecular response upon heavy metal exposure of blood on the miRNAs level may reflect the response of neurons. In that case, similar miRNAs should be identified in human brain tissue. To analyze the expression of these miRNAs in human brain tissue, we used miRNA sequencing data from Memories project, all four miRNAs were detected, while two miRNAs (miR-627 and miR-3651) were identified at the low reads’ abundance. Therefore, we checked the expression of these four miRNAs in another GEO dataset (GSE157239), which includes more samples (8 AD patients and 8 controls) from superior temporal gyrus and middle temporal gyrus. However, all four miRNAs were identified at the high abundance of reads in human brains, and two miRNAs (miR-3651 and miR-664b-3p) were significantly differentially expressed in brains of AD versus controls (p value < 0.05) (Fig. 3). Moreover, we confirmed the expression of these two differentially expressed miRNAs in serum and CSF derived from the AD subjects. The change in expression of these miRNA in human brains of AD cases vs controls conforms to the direction of change with lead exposure [downregulated in AD and negatively correlated to lead intensity (see Figs. 2 and 3)].

### Brain expressed targets of lead associated miRNAs enriched in potentially AD-related pathways

To better understand the function of these four miRNAs, we obtained their gene targets based on the experimentally validated miRNA-target interactions from the database miRTarBase^55^. A linear mixed model was utilized to identify the correlation between miRNA and its targets in blood cells. Applying an FDR cut off < 0.1, we identified 103 inversely related targets, and 252 positively related targets (Supplementary Table S3). Often the pathway analysis of potential target genes of miRNAs is highly unspecific since these targets are reported/identified for multiple tissues. Since we were interested in the targets that are truly expressed in human brains, we utilized the gene expression data from brain tissue samples of the Memories project^49^ to validate the target genes presence in human brain tissue. Of these correlated gene targets, 297 have also been expressed in human brain tissue (86 are up and 211 are downregulated).

**Figure 4:**
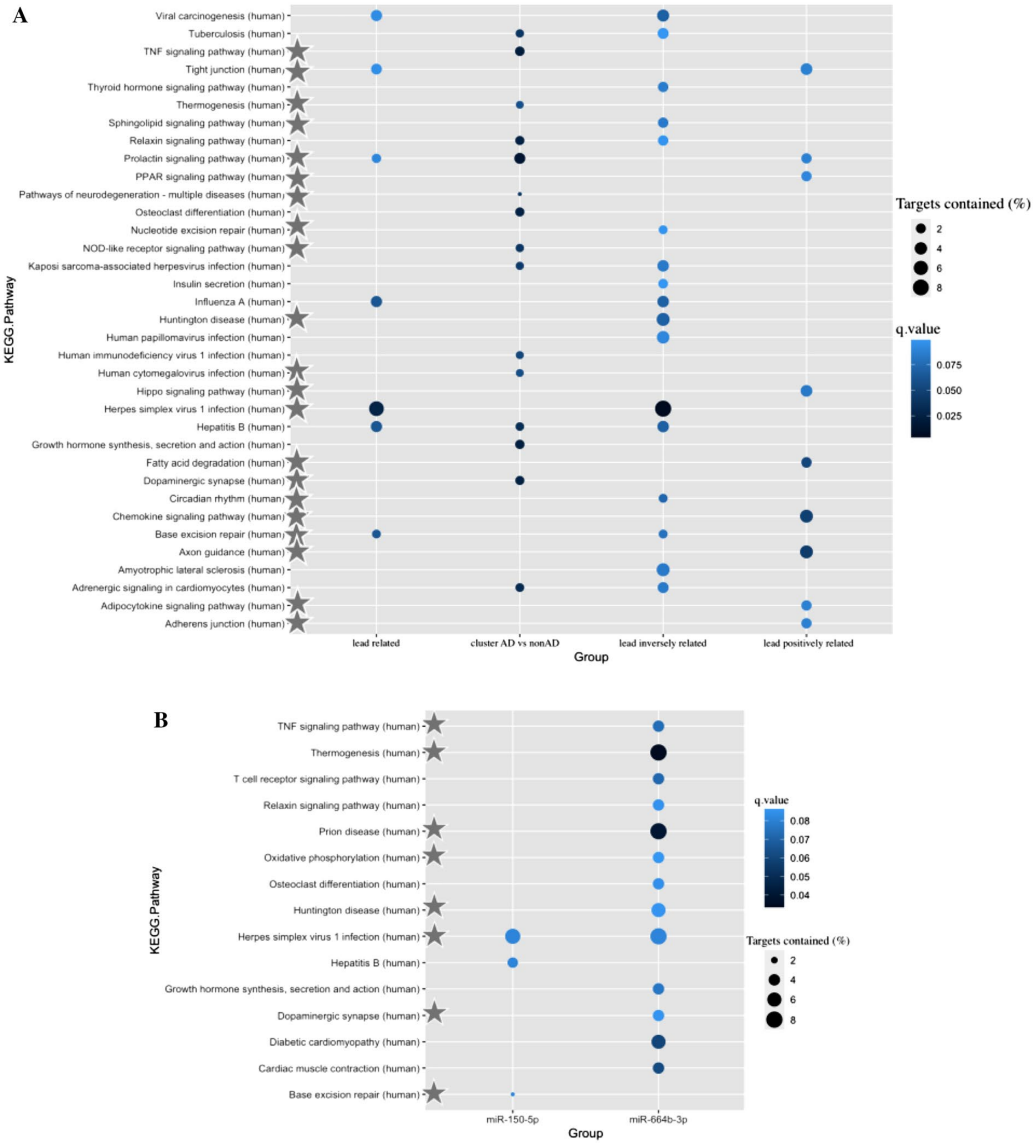
Bubble plot of enriched pathways from the miRNA-gene targets. (**A**) Pathways for gene targets that were expressed in human brain tissue of lead exposure associated miRNAs. The pathways marked with a star were potentially AD-related based on literature (Supplementary Table S1). The group lead related refers to all gene targets (associated with lead-affected miRNAs’ expression) enriched pathways, lead inversely related refers to inversely genes (positively related to miRNAs that were negatively related to lead exposure or negatively related to miRNAs that were positively related to lead exposure) enriched pathways, lead positively related refers to positively genes (positively related to miRNAs that were positively related to lead exposure or negatively related to miRNAs that were negatively related to lead exposure) enriched pathways, and cluster AD vs nonAD refers to pathways of 27 genes that were obtained via feature selection as described in Fig. 5; (**B**) Pathways for each miRNA’s gene targets that were associated with miRNA expression and expressed in human brain tissue. The pathways marked with star were potentially AD relevant according to literature (Supplementary Table S1). The targets of miR-3651 and miR-627 were not significantly enriched in any KEGG pathways.

**Figure 5:**
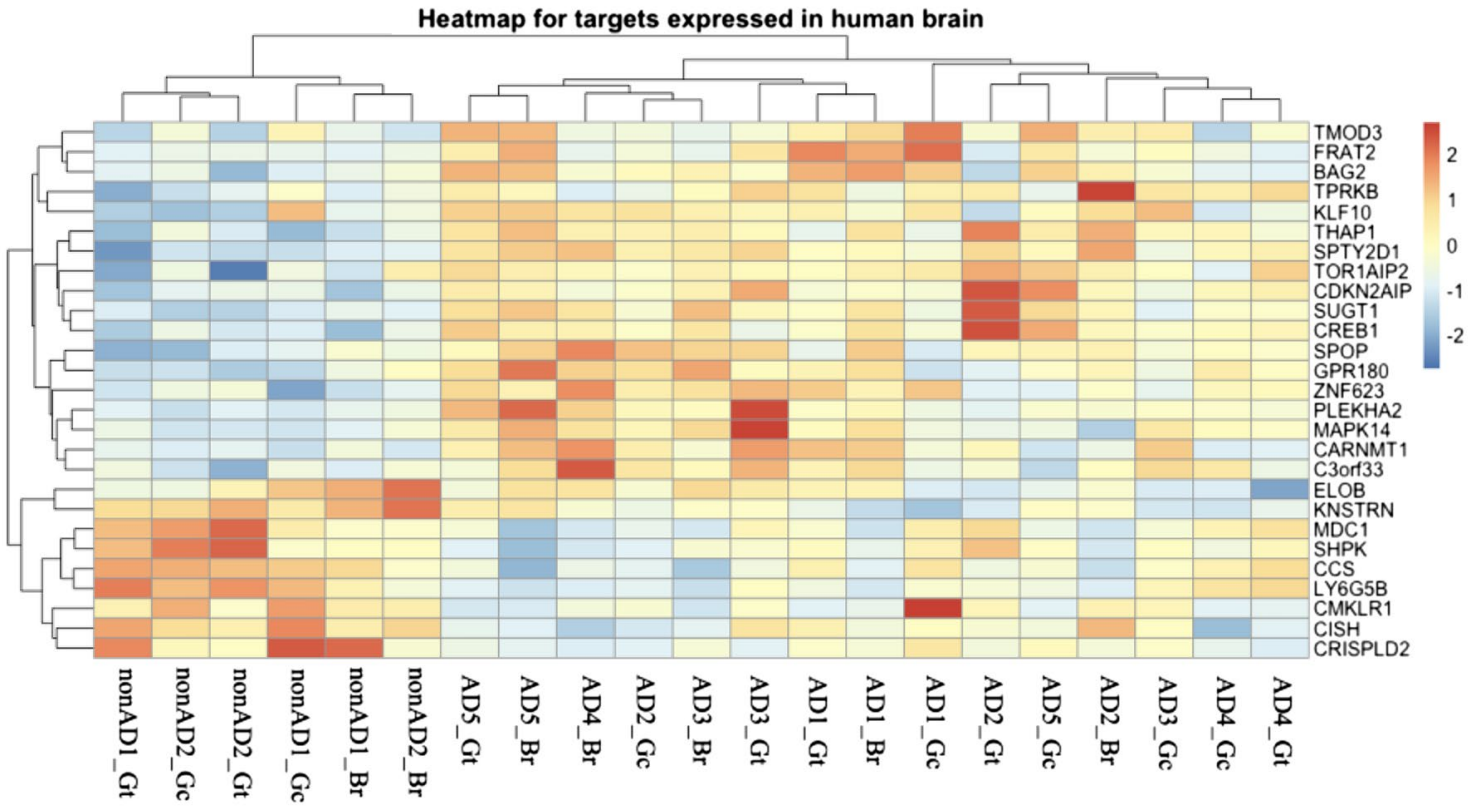
Heatmap of potentially AD-related gene targets ‘expression in three human brain regions from the Memories project. These 27 genes were obtained via feature selection based on gene-expression data of brain tissue. The expression of 27 genes in all three regions of human brain tissue from five AD cases and two controls were included, Gt, Gc and Br are the abbreviation of three regions that were described in “Materials and methods”, and clinical information for these cases were shown in Table 1.

The gene targets, of which we confirmed expression in human brain, were used to performed pathway analysis in ConsensusPathDB^60^. In our study, we defined the indirect relationship between lead exposure and gene targets’ expression as inversely and positively based on relationships between lead exposure and miRNAs’ expression, and between miRNAs’ expression and their targets’ expression. We focused on the top 35 unique significant pathways (q.value < 0.1) as shown in Fig. 4A, of which 17 pathways were significant for lead inversely related targets, nine pathways were significant for lead positively related targets, seven pathways were significant for all lead related targets (targets either inversely or positively associated were considered), and 15 pathways were significant for gene cluster AD versus non-AD that were obtained via feature selection and is further described in Fig. 5. According to literature, more than half (21/35) of the pathways were potentially AD-related (Supplementary Table S4). Within these pathways, tight junction was involved in blood–brain barrier that is important in regulating the exchange of molecules between brain and blood and maintaining brain homeostasis. Furthermore, the loss of cortical tight junction proteins is common in AD, which also correlated with synaptic degeneration^64^. Referring to synaptic function, other pathways in our study were also relevant, such as prolactin signaling pathway^65^ and chemokine signalling pathway^66^.

To investigate these four miRNAs’ potential function, we also conducted pathway analysis based on each miRNA’s gene targets, which were associated with miRNA expression and expressed in human brain tissue (Fig. 4B). Only the identified target genes of miR-150-5p and miR-664b-3p were significantly (q value < 0.1) enriched in KEGG pathways (3 and 13 respectively), and one overlapping pathway (herpes simplex virus 1 (HSV-1) infection) was found. Overall, 8/15 pathways were potentially AD relevant according to literature (Supplementary Table S4).

### Brain expressed AD relevant targets enriched in potentially AD-related pathways

From the Memories project gene-expression dataset^49^, we identified gene targets of the four lead exposure associated miRNAs and 786 gene targets were expressed in all three human brain regions. Feature selection was performed based on elastic net regression to recognize AD relevant gene targets. Finally, 27 gene targets were selected as AD relevant (Supplementary Fig. S4). The heatmap of these genes showed that AD cases were clustered together (Fig. 5). Here, we also found heterogeneous gene expression in different regions of same candidate. For AD cases, the expression of these 27 genes was more irregular. However, the heatmap still reflected clinical-related differences, for example, case AD5 was far away from case AD3, as shown in Table 1, the Braak stage of AD5 was III after 1 year diagnosis for AD, while the Braak stage of AD3 was IV after four years duration. AD2 had most heterogeneous gene expression with three brain regions distributed in different clusters, and this case had the highest Braak stage V in this group of AD patients. We also checked the clinical-related difference in miRNAs data (Supplementary Fig. S5), in which AD3 is also far away from AD5, and AD1 and AD3 are even clustered in the control group, both AD cases were following the disease progression of increasing one Braak stage each year. Further studies including more samples should be developed to examine this clinical-related difference among genes.

The 27 gene targets were enriched in 15 significant KEGG pathways (q.value < 0.1) (Fig. 4A: the group cluster AD vs nonAD), of which seven pathways were potentially AD related (Supplementary Table S4) such as the pathway of neurodegeneration^67^.

### The interaction network of miRNAs, TFs and gene targets is linked to AD

To better understand the complex regulations between miRNAs and gene targets, we also included analysis of TFs. TFs are the most important trans-acting factors involved in transcriptional regulation, which bind to cis-regulatory elements of the DNA and activate RNA polymerase to begin the transcription of target genes^58^. The expression of miRNAs can be activated or repressed by TFs, in addition, miRNAs and TFs may cooperate to tune gene expression^68^. We obtained the TFs-miRNAs regulations from TransmiR v2^57^ and TFs-targets regulations from TRRUST v2 database^58^ respectively.

**Figure 6:**
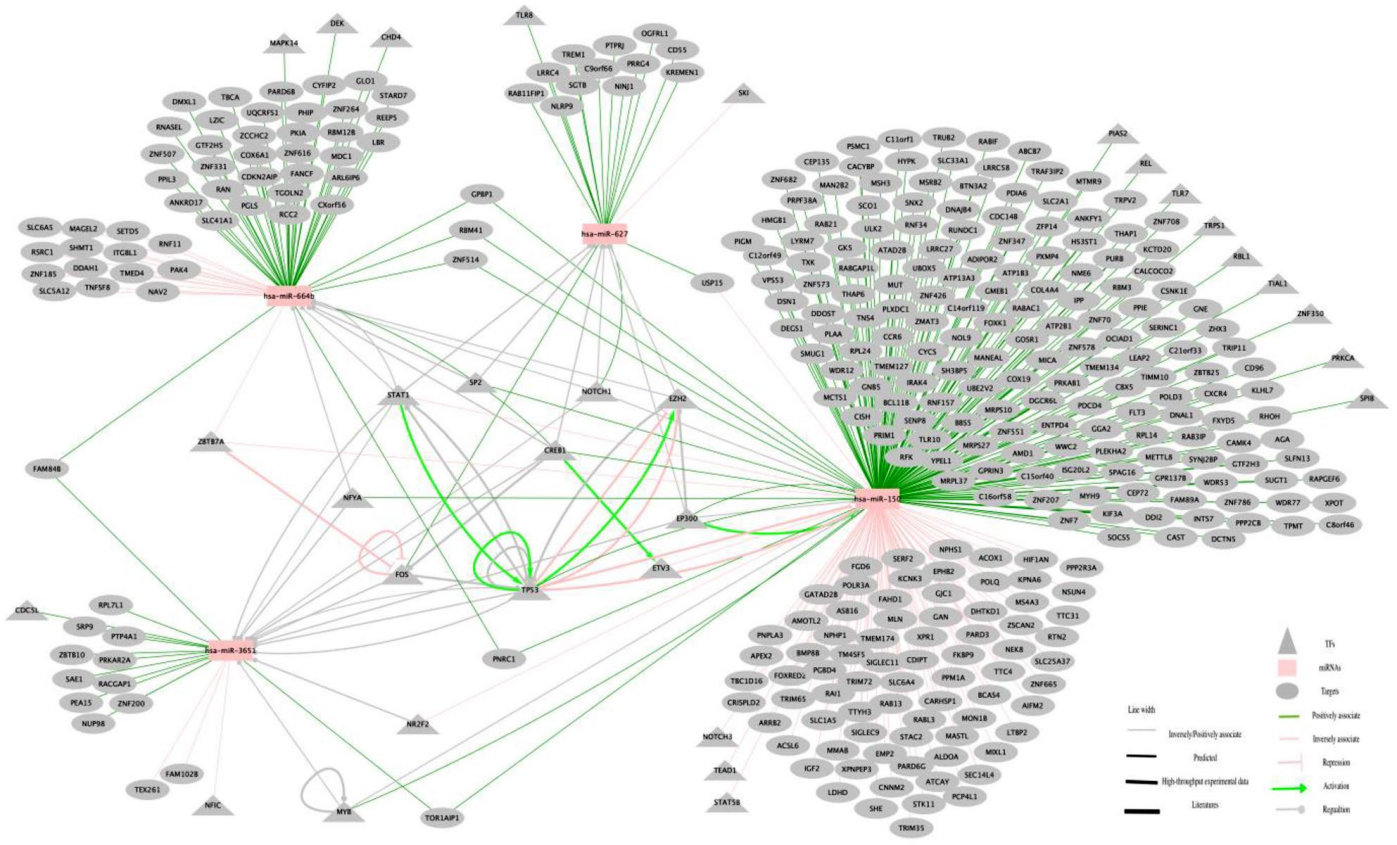
The co-regulation of the complex interaction of miRNAs, TFs and gene targets based on data integration from different data sources. This figure was made using Cytoscape. For the four miRNAs, they were linked to their targets in this network based on coefficients of linear mixed model (Supplementary Table S3), some targets are TFs. In addition, the TFs-miRNAs regulations from TransmiR v2 database^57^ and TFs-targets regulations from TRRUST v2 database^58^ were also visualized based on evidence level (Supplementary Table S5). In total, there are 13 TFs became hubs of the network including ZBTB7A, MYB, NR2F2, FOS, NFYA, STAT1, SP2, CREB1, ETV3, EZHZ, EP300, TP53 and NOTCH1. Detailed interact information was described in Supplementary Tables S3 and S5.

Among the gene targets of the four lead exposure associated miRNAs, 353 targets were unidirectional interactions and one was bidirectional (USP15). According to TransmiR v2 and TRRUST v2 databases, 32 are TFs, and 20 TFs are potentially involved in AD pathology (Table 2), 13 TFs become hubs in the network (Fig. 6). Based on the validation dataset which is the brain tissue gene-expression data from samples described in Table 1, 13/20 TFs are expressed in human brains and 7/13 TFs show consistent expression difference in all three brain regions in AD cases versus healthy controls (Supplementary Fig. S6).

**Table 2:** The potentially AD-related TFs among gene targets of lead exposure associated miRNAs based on TransmiR v2 and TRRUST v2 databases.

TFs	Interact with (miRNAs in our analysis)	Involved in AD study	Supported reference	Detected in human brain tissue? (Y or –)	Expressed in all three brain regions with consistent direction? (Y or –)
**STAT5B**	hsa-miR-150-5p	Modulates learning and memory formation	^86^	Y	–
**EP300**	hsa-miR-150-5p, hsa-miR-3651, hsa-miR-627	Tau modifiers	^87^	Y	Y
**STAT1**	hsa-miR-150-5p, hsa-miR-3651, hsa-miR-627, hsa-miR-664b-3p	Negatively regulate spatial learning and mediate the memory-impairing effect of Aβ	^88^	Y	Y
NFIC	hsa-miR-3651	Identified as novel loci in the AD	^89^	Y	Y
**FOS**	hsa-miR-627, hsa-miR-3651	Apoptotic gene, was up-regulated expressed in AD	^90^	–	–
**CREB1**	hsa-miR-150-5p, hsa-miR-3651, hsa-miR-627, hsa-miR-664b-3p	Involved in synaptic plasticity that mediates the conversion of short-term memory to long-term memory	^91^	Y	Y
NOTCH3	hsa-miR-150-5p	Identify pathogenic mutation in AD	^92^	–	–
REL	hsa-miR-150-5p	Elicite neuroprotection by activation of metabotropic glutamate receptors type 5 (mGlu5) and finally be against Aβ toxicity	^93^	Y	Y
TLR7	hsa-miR-150-5p	Present in neurons, and its activation by let-7 initiated neurodegeneration	^94^	–	–
MAPK14	hsa-miR-664b-3p	target innate immune responses in the brain and reduce inflammation-induced synaptic toxicity	^95^	Y	Y
PRKCA	hsa-miR-150-5p	Exert an effect through hsa-miR-146a regulation in AD	^96^	Y	–
CDC5L	hsa-miR-3651	Dysregulated, have function on cell apoptosis	^97^	Y	–
TLR8	hsa-miR-627	RNA from an HERV-K(HML-2) envelope gene region binds to and activates human TLR8, expressed in neurons and microglia, thereby causing neurodegeneration	^98^	–	–
**SP2**	hsa-miR-150-5p, hsa-miR-627, hsa-miR-664b-3p	A cell cycle regulator, deletion of which disrupts neurogenesis in embryonic and postnatal brain	^99^	Y	Y
CHD4	hsa-miR-664b-3p	Activity-dependent neuroprotective protein, via the recruitment of HP1 and CHD4, regulates the expression of genes that are crucial for maintaining distinct cellular states and assures accurate cell fate decisions upon external cues	^100^	Y	–
**NOTCH1**	hsa-miR-150-5p, hsa-miR-3651, hsa-miR-627, hsa-miR-664b-3p	affects synaptic plasticity, memory and olfaction	^101^	–	–
DEK	hsa-miR-664b-3p	DEK loss in vitro recapitulates cellular and molecular phenotypes of AD pathology	^102^	Y	–
**MYB**	hsa-miR-150-5p, hsa-miR-3651	Involved in protection for Aβ toxicity and in neuronal survival	^103^	–	–
SPIB	hsa-miR-150-5p	The binding site is the regulator of AD during apoptosis pathway and inducing cell death and apoptosis	^104^	–	–
TP53	hsa-miR-150-5p, hsa-miR-3651	Mutations in exon 7 of TP53 (C748A, C708T) may be associated with pathogenesis of AD	^105^	Y	–

The bold TFs are hubs in the network (Fig. 6), “Y” means yes, “–” means no. The supported reference was the most relevant literature.

These complex interactions are visualized in a network (Fig. 6), where co-regulations among lead-associated miRNAs, TFs and respective gene targets (Supplementary Table S5) are shown. The TFs that were present as hubs of the network might play a role in the mechanism of lead exposure and AD.

## Discussion

In this study, we explored the impact of lead exposure on miRNA expression from 186 buffy coat samples of healthy subjects, with normal lead-exposure for the general population at that time. We explored how lead exposure associated miRNAs function as biomarkers in relation to AD and AD processes. Four miRNAs were identified as lead exposure associated, and these were all detectable in human brain tissue according to the publicly available dataset GSE157239. Moreover, two of these miRNAs showed differential expression in the brain of AD versus controls in accordance with the direction of change upon lead exposure. Their gene targets were analyzed, and 253 targets were positively related to miRNAs, while 103 were inversely related. Among these targets, 297 are found to be expressed in brain tissue with 86 being up and 211 being downregulated. Furthermore, pathway analysis showed that these targets were enriched in numerous AD-related pathways (Fig. 4). We identified TF-miRNAs and TF-gene target pairs which were also involved in AD processes (Fig. 6).

Among the four lead associated miRNAs, miR-150-5p was negatively related to lead exposure, and it was previously found down-regulated in AD in CSF^69^. Moreover, in our study we identified a significant negative correlation between miR-150-5p and its target gene SLC1A5, which was identified to affect the response to prenatal lead exposure on neurodevelopmental outcomes in a previous genome-wide gene-environment interaction study^70^. The gene SLC1A5 was involved in synaptic function, neuronal development, and excitotoxicity^70^. In our analysis, SLC1A5 was positively associated with lead exposure and negatively associated with miR-150 suggesting that this miRNA is also involved in processes by which lead affects neurodevelopment and AD development.

As shown in Fig. 6, these four miRNAs were all correlated with STAT1 that is a TF regulating the expression of BACE1. Recently, a predictive model consisting of five BACE1-related plasma miRNAs was developed as a novel biomarker for the prodromal stage of AD^71^. Though the four miRNAs we found in this study did not target BACE1 directly, they all associated with STAT1 that regulates BACE1, therefore there might be a potential connection. MitomiRs are a subset of miRNAs found to be localized to human mitochondria and potentially regulate mitochondrial function under the physiological and pathological conditions of AD^72^, interestingly 3/4 miRNAs (miR-3651, miR-664b-3p and miRNA-150-5p) in our study were all mitomiRs^73–75^, which might suggested a potential role of these miRNAs in AD.

Cadmium and lead concentrations from blood samples of healthy people were measured, but only lead exposure was considered since no miRNAs were associated with cadmium level. However, cadmium exposure does play a role in miRNA expression. In a previous study, miR-122-5p and miR-326-3p were found consistently overexpressed in cell lines and different types of bio-samples of rats and humans exposed to cadmium^76^. Only miR-326 was detected in our study but showed no significant association with cadmium. A reason for these inconsistent results might be varying intensity of cadmium exposure between our study and the previous study, where occupational exposure to cadmium was considered, which usually means that their study subjects have higher exposure to cadmium, while this was not the case in our study. A consequence of our study design is that the four lead related miRNAs should be considered of relevance at the low-level lead exposure that occurs in the general population, that is they represent mechanisms that are probably operating in our bodies not only at unusually elevated exposures but under ‘normal’ conditions.

We conducted pathway analysis using brain expressed gene targets of these lead exposure associated miRNAs, and many of the pathways were AD-relevant according to the literature. These pathways were involved in different mechanisms, such as synaptic function, neuron death and blood–brain barrier. For example, axon guidance is a crucial process for neural circuit formation^77^ which is an important topic in AD study since it remains unresolved why neuronal circuits become dysfunctional in response to high Aβ levels and how circuit abnormalities could be repaired^78^. Aβ and hyperphosphorylation of tau could cause synaptic damage, leading to AD, while axon-guidance molecules could regulate the balance between synapse formation and Aβ^79^. Within this study, four genes belonged to this pathway, and they were regulated by miR-150-5p, miR-627 and miR-664b-3p. Therefore, lead exposure might affect the expression of miRNAs and gene targets, impairing axon guidance function, finally causing failure to regulate Aβ and synapse formation. As lead is mainly known for its effects on neural development in fetuses and children, it is worth noting that these mechanisms might be of relevance also to the neurodevelopmental effects of lead. Additionally, the targets of miR-664b-3p and miR-150-5p were both enriched in HSV-1 infection. This pathway has attracted a lot of attention on AD development, since this virus could initiate an innate immune system response that causes the increased production of Aβ, and this increased production subsequently causes neuroinflammation. Inflammation could lead to damage to the brain cells, eventually resulting in dementia^80^.

Furthermore, the pathways which were also linked to lead exposure might provide better understanding in how these miRNAs were involved in relating lead exposure with AD. As shown in Fig. 4A, prolactin signaling pathway was enriched in both lead exposure relevant targets and AD relevant targets of our four miRNAs. The prolactin level has been altered in both AD patients^81^ and in workers chronically exposed to lead^82^. One potential process is that lead influences the dopaminergic system, which controls prolactin secretion within the pituitary gland, while the prolactin has inflammatory and anti-inflammatory effects via activating or inactivating different signaling pathways, with inactivating GSK3 it can inhibit tau phosphorylation^65^.

The analysis of TFs provides another approach to understanding the connections between lead exposure and AD. In the network involving miRNAs, TFs, and gene targets, the hub genes might play an important role in linking lead exposure and AD. For example, STAT1, lead could decrease the ipopolysaccharide-induced expression of the STAT1 which is involved in transcription of inducible nitric oxide synthase (iNOS)^83^, and iNOS has been studied as a major instigator of Aβ deposition and disease progression^84^. SP2 is also found to be lead relevant because lead substitution in Sp1 could cause the upregulation of APP transcription, then cause the build-up of Aβ in the brain and contribute to AD, and it has been known that the Sp-family of TFs (Sp1, Sp2, Sp3, and Sp4) regulate the same genes^85^. Even though the regulation among miRNAs, TFs, and gene targets were quite complex as shown in Fig. 6, we still recognized some TFs that link to both AD and lead exposure, and further study is needed to reveal the specific mechanisms.

However, there are some limitations in this study. When validating the expression of miRNAs in human brain, the number of samples that we used is limited. Via cluster analysis as presented in Figs. 5 and S5, we found clinical-related differences due to the different AD stages of the subjects, this finding requires validation in further studies with larger sample sizes. Besides, the clinical information of GSE157239 was limited and we were not able to check the clinical-related differences in relationship to the expression of miRNAs as we shown in Fig. 5. Also, we confirmed the expression of the differentially expressed miRNAs on CSF and serum, however only for AD cases not for controls. Future study with larger sample size would be needed to follow up on the potential of these miRNAs as biomarkers for AD.

In the present study, we investigated miRNA expression in a human population with known blood-lead concentrations. We identified four miRNAs (hsa-miR-3651, hsa-miR-150-5p, hsa-miR-664b-3p and hsa-miR-627) that were lead exposure related at the low exposure levels of the population, and demonstrated their potential involvement in AD development. Moreover, their targets were found enriched in potential AD-related pathways, and some of these targets represent AD pathology associated TFs, but may also be involved in earlier neurodevelopment, for example during fetal life. Further studies are needed to confirm the involvement of these miRNAs in lead toxicity as well as in AD development. Nevertheless, our data identified new miRNAs as lead exposure related, and these miRNAs show potential as new biomarkers for the identification of human populations exposed to heavy metals with a significant risk of developing AD.

## Supplementary Information

Supplementary Information.

## Data Availability

The dataset from the EnviroGenomarkers project is not publicly available due to restrictions imposed by Swedish legislation on the protection of personal data. Requests to access the datasets should be directed to Ingvar A. Bergdahl, ingvar.bergdahl@umu.se. Sequencing data from the Memories project is available upon reasonable request but clinical information is not publicly available due to restrictions imposed by the Belgian/Dutch legislation on the protection of personal data. Request to access the data should be directed to Sebastian Engelborghs, sebastiaan.engelborghs@uantwerpen.be.
