# Supplementary material for: Lead-exposure associated miRNAs in humans and Alzheimer’s disease as potential biomarkers of the disease and disease processes
Source: Sci Rep. 2022 Sep 24;12:15966. doi: 10.1038/s41598-022-20305-5 (PMC9509380; doi:10.1038/s41598-022-20305-5)
Supplement: Supplementary file 1 — Supplementary Information. [file 41598_2022_20305_MOESM1_ESM.docx]

**Supplementary Figure S1.** The QQ plots of confounders.

**
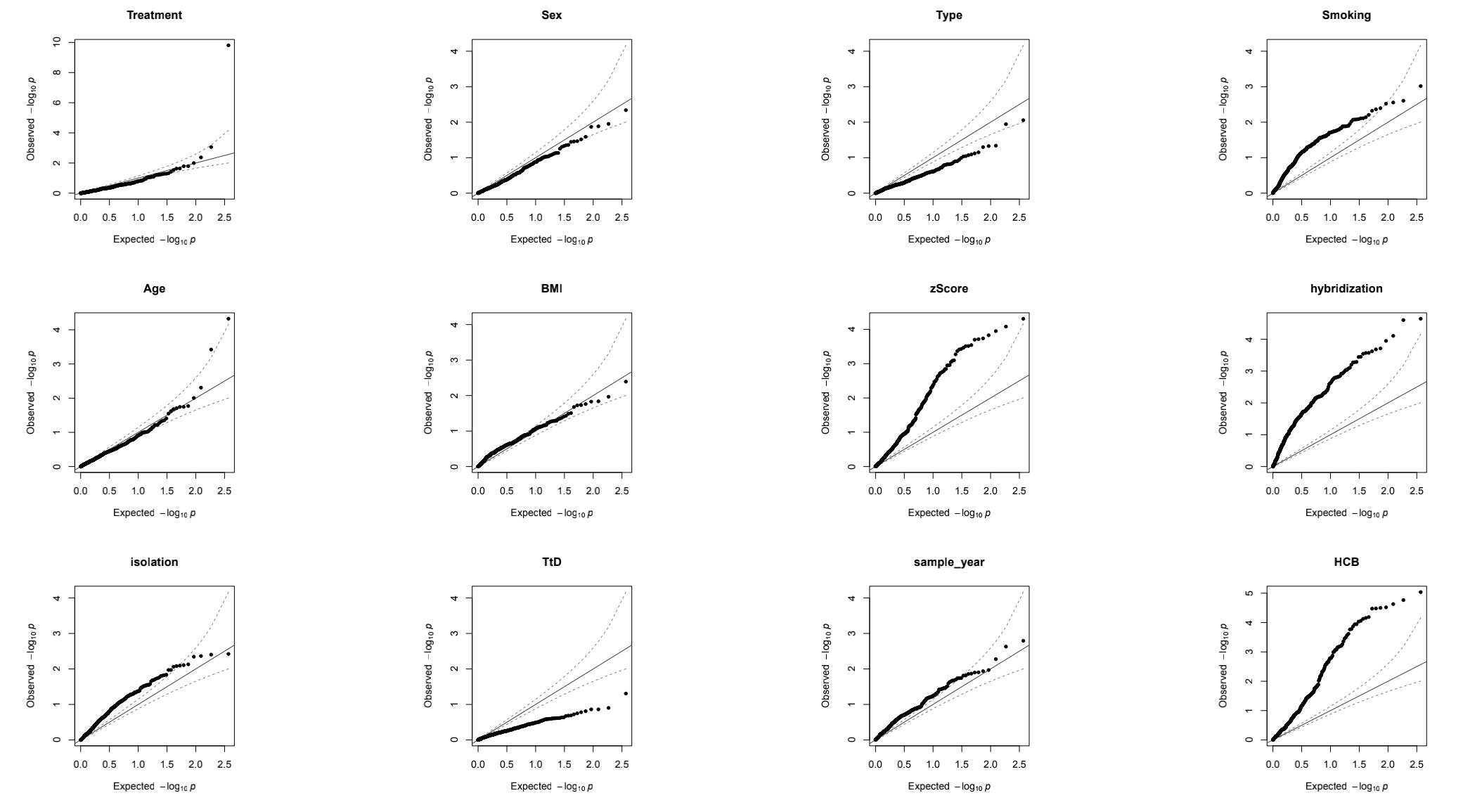
**

**
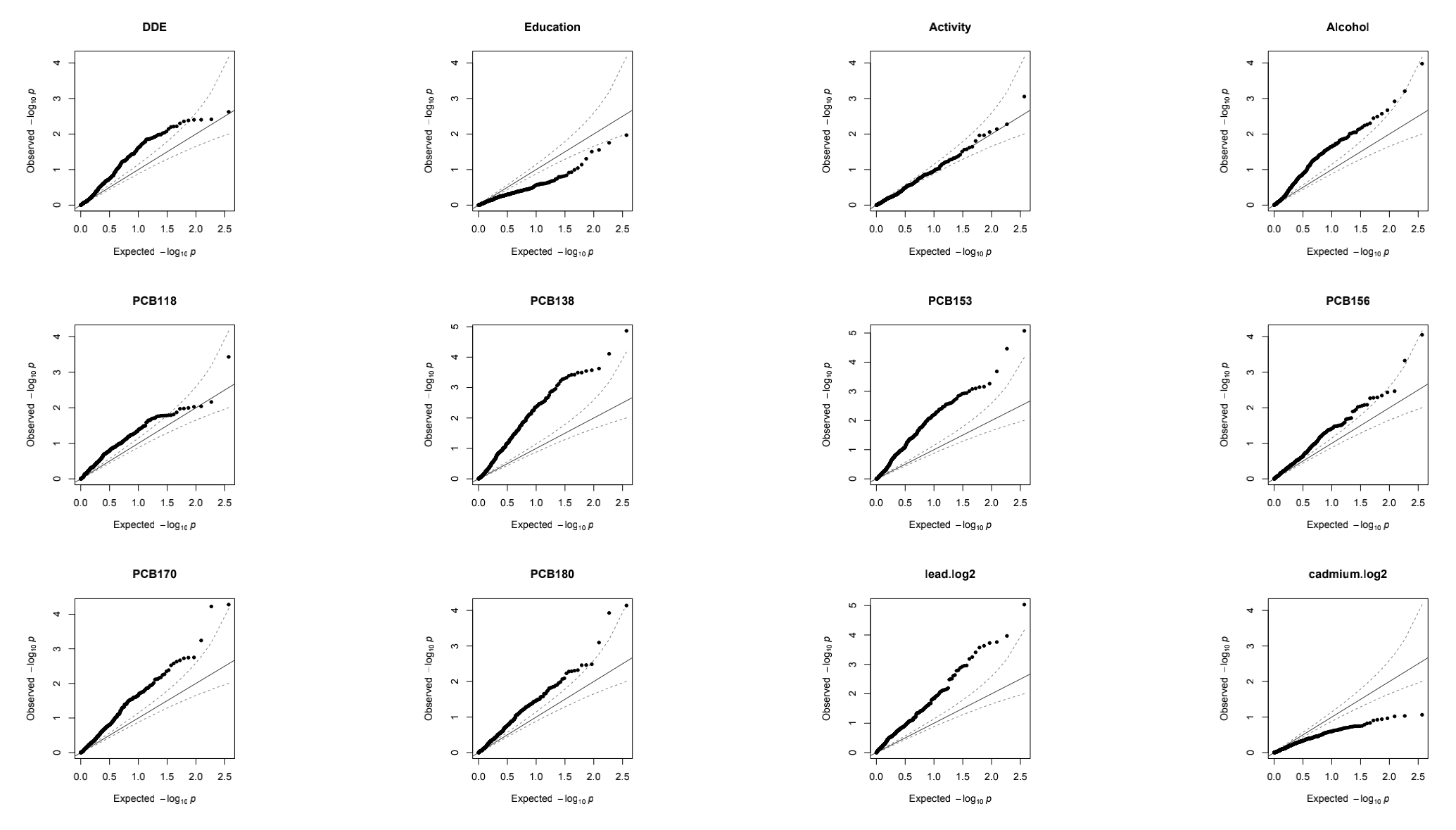
**

**Supplementary Figure S2**. Feature selection for miRNAs via glmnet package in R software. When we set alpha=0.5 (elastic net regression algorithm), mean-squared error was least, which is the b plot. In this study, we selected 26 miRNAs as important lead exposure related miRNAs to do further analysis.


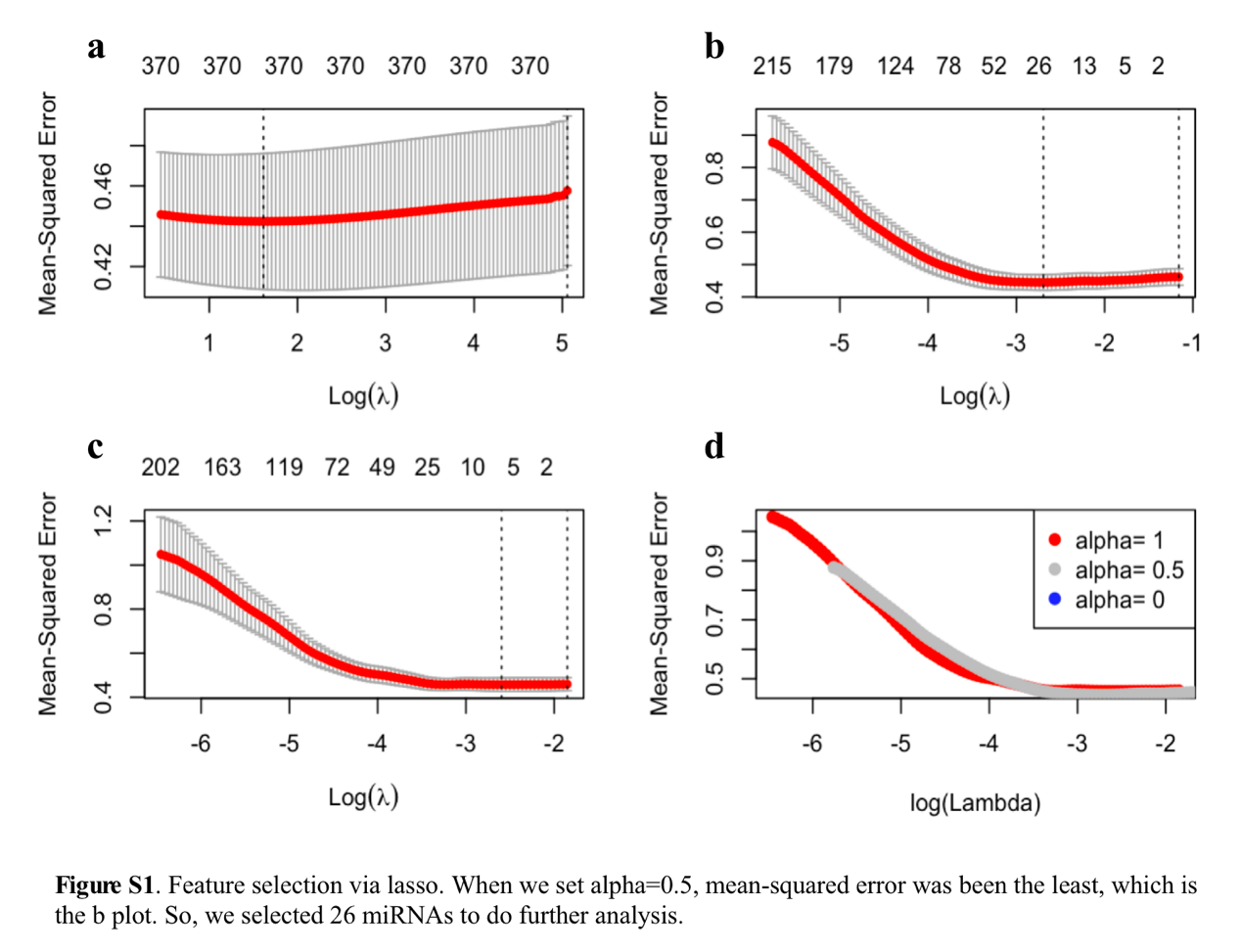


**Supplementary Figure S3.** The correlation among covariates based on Pearson Correlation Coefficients.


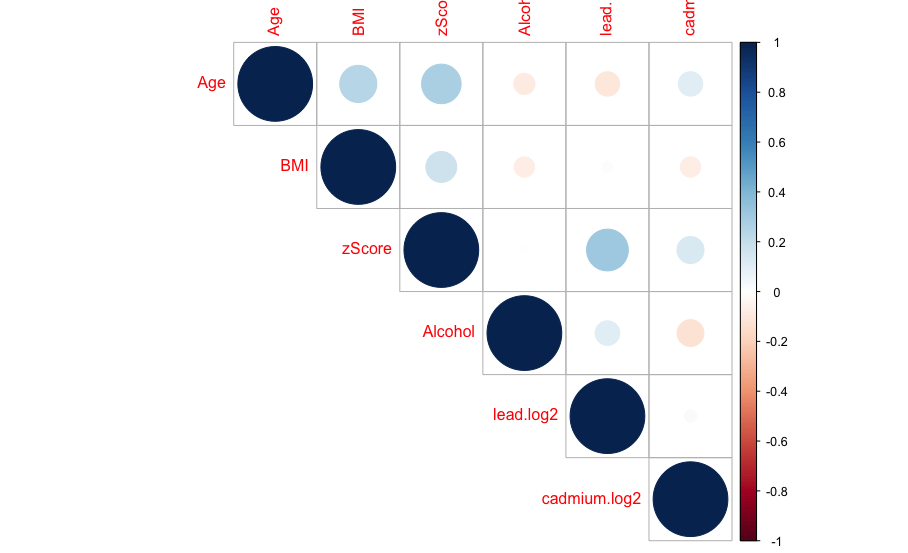


**Supplementary Figure S4.** Feature selection for gene targets via glmnet package in R software. When we set alpha=0.5 (elastic net regression algorithm), misclassification error was least, which is the b plot. Here, we selected 27 gene targets as important AD related targets.


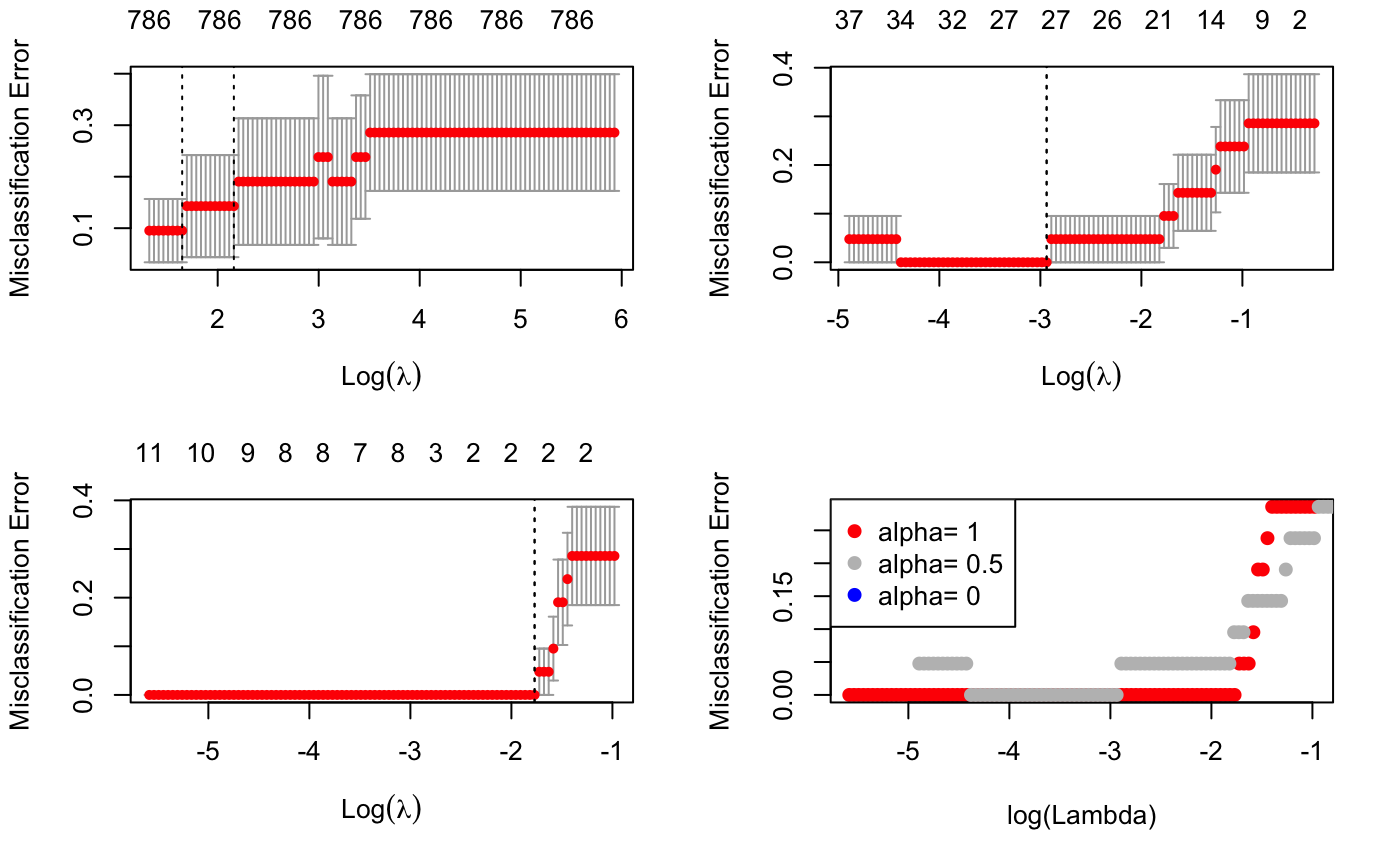


Supplementary Figure S5. Heatmap of lead exposure associated miRNAs‘ expression in three human brain regions from the Memories project. These two miRNAs were identified with high abundance of reads in all three regions of human brain tissue from five AD cases and two controls. Gt, Gc and Br are the abbreviation of three regions that were described in Methods, and clinical information for these cases were shown in Table 1.


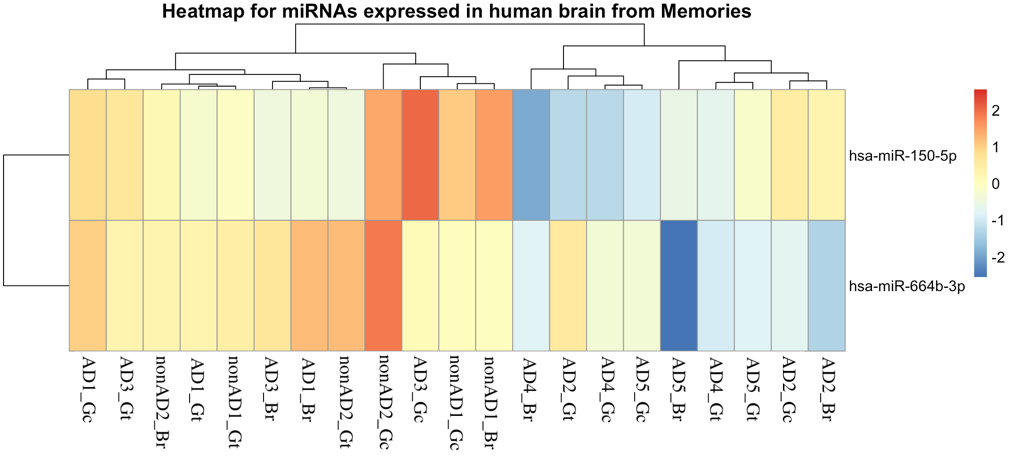


Supplementary Figure S6. The seven TFs expression value in three brain regions in AD cases versus healthy controls. The width of box is based on sample size. The p-values were obtained from differential expression analysis by limma package in R software.


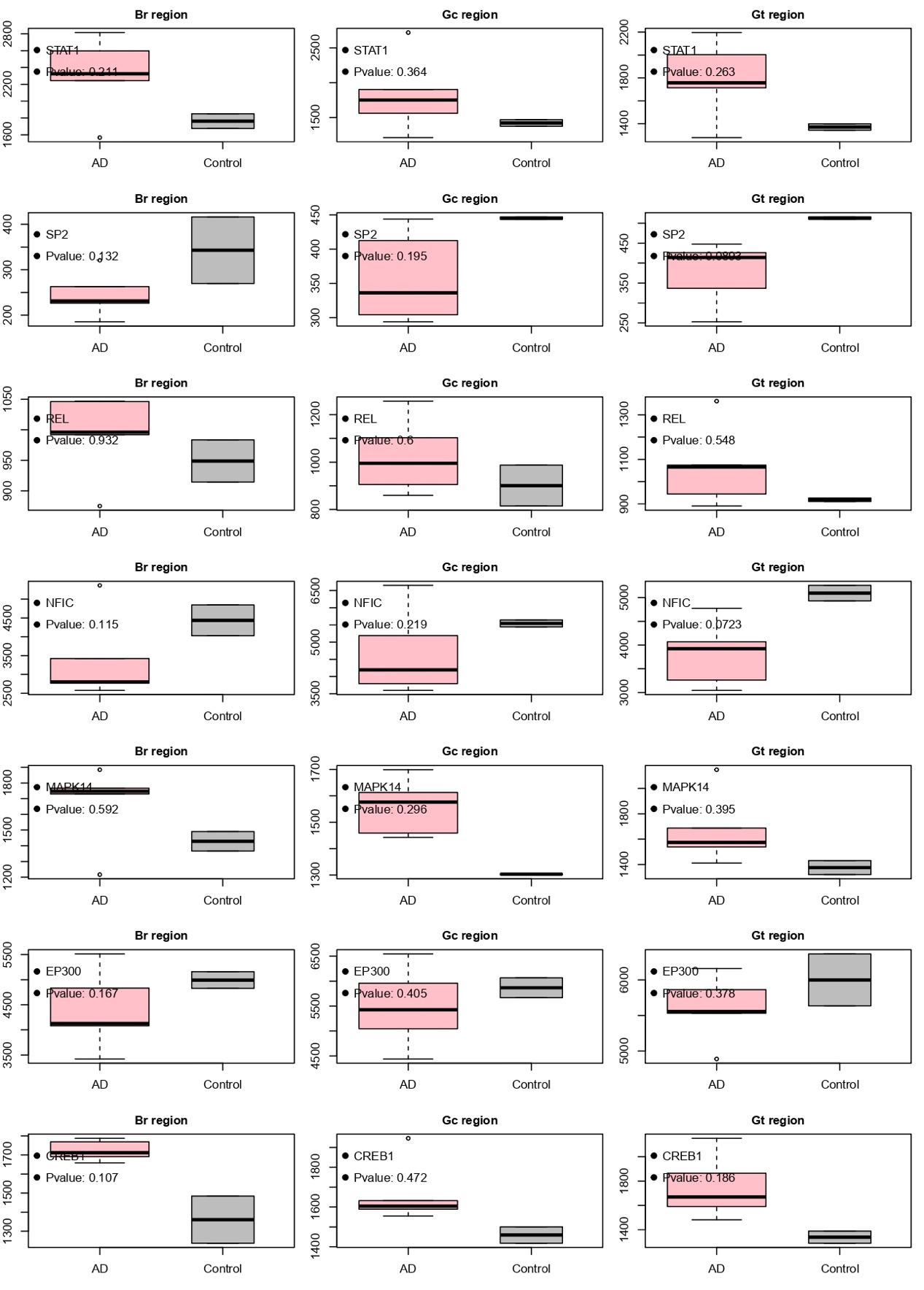


**Supplementary Table S1.** The information of GSE157239. Publicly available miRNA microarray data GSE157239 was used as validation set to study the expression of lead exposure associated miRNAs in post-mortem human brains comprising the superior and middle temporal gyrus of eight AD cases vs eight controls. We used keyword “miRNA and Alzheimer” in GEO DataSets to search for available dataset and selected only miRNA profiling data of human brain tissue. Based on sample size, GSE157239 was chosen as validation data for miRNA expression in human brain. The clinical information of GSE157239 was followed.

| **Case** | **Sex** | **Death Age** | **Braak stage** | **Control** | **Sex** | **Death Age** | **Braak stage** |
| --- | --- | --- | --- | --- | --- | --- | --- |
| AD_1 | M | 80 | Above III | CN_1 | F | 90 | - |
| AD_2 | F | 86 | Above III | CN_2 | F | 81 | - |
| AD_3 | F | 83 | Above III | CN_3 | M | 83 | - |
| AD_4 | F | 84 | Above III | CN_4 | F | 69 | - |
| AD_5 | M | 72 | Above III | CN_5 | M | 82 | - |
| AD_6 | F | 94 | Above III | CN_6 | F | 78 | - |
| AD_7 | F | 77 | Above III | CN_7 | M | 86 | - |
| AD_8 | F | 62 | Above III | CN_8 | F | 70 | - |

**Supplementary Table S2**. The information of miRNAs affected by each covariate.

| **Covariate** | **Percentage (p-value < 0.05)** | **Percentage (FDR < 0.05)** |
| --- | --- | --- |
| Sex | 3.5135135 | 0 |
| Smoking | 24.0540541 | 0 |
| Age | 4.0540541 | 0.2702703 |
| BMI | 4.0540541 | 0 |
| zScore | 21.8918919 | 10.8108108 |
| hybridization | 42.972973 | 21.6216216 |
| isolation | 12.1621622 | 0 |
| sample_year | 8.9189189 | 0 |
| HCB | 28.1081081 | 15.1351351 |
| DDE | 15.4054054 | 0 |
| Education | 1.3513514 | 0 |
| Activity | 4.5945946 | 0 |
| Alcohol | 19.1891892 | 0.2702703 |
| PCB118 | 11.0810811 | 0 |
| PCB138 | 27.8378378 | 11.6216216 |
| PCB153 | 27.8378378 | 7.027027 |
| PCB156 | 12.7027027 | 0.2702703 |
| PCB170 | 18.3783784 | 0.5405405 |
| PCB180 | 14.0540541 | 0.5405405 |
| lead.log2 | 20.2702703 | 4.0540541 |
| cadmium.log2 | 0 | 0 |

**Supplementary Table S3.** The correlation of these four miRNAs and their gene targets. The coefficient value < 0 means negative relation, and > 0 means positive relation.

| **miRNA** | **Target** | **Coefficient** | **FDR** |
| --- | --- | --- | --- |
| hsa-miR-3651 | SAE1 | 0.22509177 | 0.0000014 |
| hsa-miR-3651 | RPL7L1 | 0.14015429 | 0.00253013 |
| hsa-miR-3651 | ZBTB10 | 0.232468129 | 0.002234592 |
| hsa-miR-3651 | FAM84B | 0.359839575 | 0.004557141 |
| hsa-miR-3651 | RACGAP1 | 0.27059826 | 0.000000213 |
| hsa-miR-3651 | FAM102B | -0.469417541 | 0.0000014 |
| hsa-miR-3651 | TEX261 | -0.268300731 | 0.000592181 |
| hsa-miR-3651 | NFIC | -0.417234644 | 0.0000169 |
| hsa-miR-3651 | CDC5L | 0.149670996 | 0.0000249 |
| hsa-miR-3651 | ZNF200 | 0.13018988 | 0.01546321 |
| hsa-miR-3651 | SRP9 | 0.18624728 | 0.00012939 |
| hsa-miR-3651 | PTP4A1 | 0.183366484 | 0.00087554 |
| hsa-miR-3651 | NUP98 | 0.074195314 | 0.046225666 |
| hsa-miR-3651 | TOR1AIP1 | 0.133589219 | 0.002109987 |
| hsa-miR-3651 | PEA15 | 0.124829852 | 0.046567233 |
| hsa-miR-3651 | PRKAR2A | 0.157592614 | 0.00399652 |
| hsa-miR-150-5p | MYB | 0.171109622 | 0.00000144 |
| hsa-miR-150-5p | IGF2 | -0.545888791 | 0.00000401 |
| hsa-miR-150-5p | CXCR4 | 0.202435502 | 0.000146689 |
| hsa-miR-150-5p | NOTCH3 | -0.445719206 | 1.06E-08 |
| hsa-miR-150-5p | FLT3 | 0.279810846 | 0.0000219 |
| hsa-miR-150-5p | EP300 | 0.135399103 | 0.000225069 |
| hsa-miR-150-5p | MS4A3 | -0.139648248 | 0.029185387 |
| hsa-miR-150-5p | AGA | 0.160807824 | 0.00007 |
| hsa-miR-150-5p | ATP13A3 | 0.130426372 | 0.039876155 |
| hsa-miR-150-5p | TP53 | 0.235574881 | 0.004154317 |
| hsa-miR-150-5p | ADIPOR2 | 0.090529076 | 0.001194551 |
| hsa-miR-150-5p | ATP2B1 | 0.087357434 | 0.02089757 |
| hsa-miR-150-5p | PURB | 0.057925706 | 0.006723606 |
| hsa-miR-150-5p | FOXK1 | 0.081609099 | 0.015984017 |
| hsa-miR-150-5p | ZBTB7A | -0.111747439 | 0.000914547 |
| hsa-miR-150-5p | PDIA6 | 0.13569233 | 0.000621514 |
| hsa-miR-150-5p | TRPS1 | 0.158763358 | 0.000107947 |
| hsa-miR-150-5p | CAST | 0.170227397 | 0.000000174 |
| hsa-miR-150-5p | AIFM2 | -0.573394802 | 6.57E-10 |
| hsa-miR-150-5p | COL4A4 | 0.421355598 | 0.000000121 |
| hsa-miR-150-5p | CISH | 0.162284721 | 0.041185046 |
| hsa-miR-150-5p | CCR6 | 0.25588401 | 0.00000146 |
| hsa-miR-150-5p | WDR77 | 0.138877807 | 0.000000017 |
| hsa-miR-150-5p | MRPS27 | 0.305414417 | 0.000000173 |
| hsa-miR-150-5p | GAN | -0.109088141 | 0.046578261 |
| hsa-miR-150-5p | DGCR6L | 0.06120503 | 0.063424959 |
| hsa-miR-150-5p | XPR1 | -0.233166285 | 0.0000124 |
| hsa-miR-150-5p | ABCB7 | 0.212174805 | 4.12E-10 |
| hsa-miR-150-5p | ACSL6 | -0.115499753 | 0.015604939 |
| hsa-miR-150-5p | SMUG1 | 0.086035571 | 0.006664531 |
| hsa-miR-150-5p | PPM1A | -0.276865292 | 5.75E-09 |
| hsa-miR-150-5p | BMP8B | -0.120525965 | 0.007642798 |
| hsa-miR-150-5p | SERF2 | -0.161465908 | 2.29E-09 |
| hsa-miR-150-5p | FXYD5 | 0.0822256 | 0.000543249 |
| hsa-miR-150-5p | SIGLEC11 | -0.185628951 | 0.044656576 |
| hsa-miR-150-5p | IPP | 0.343653011 | 3.14E-13 |
| hsa-miR-150-5p | BCAS4 | -0.214352437 | 0.001520252 |
| hsa-miR-150-5p | MIXL1 | -0.121849291 | 0.004623431 |
| hsa-miR-150-5p | ZNF708 | 0.127225438 | 0.002806276 |
| hsa-miR-150-5p | COX19 | 0.143021317 | 0.00000889 |
| hsa-miR-150-5p | FGD6 | -0.076043112 | 0.052478122 |
| hsa-miR-150-5p | SOCS5 | 0.241575705 | 3.23E-10 |
| hsa-miR-150-5p | HYPK | 0.236872234 | 1.16E-10 |
| hsa-miR-150-5p | GGA2 | 0.311181037 | 3.5E-14 |
| hsa-miR-150-5p | CYCS | 0.172457819 | 0.0000316 |
| hsa-miR-150-5p | CACYBP | 0.164331555 | 0.000000434 |
| hsa-miR-150-5p | NME6 | 0.058367571 | 0.01205419 |
| hsa-miR-150-5p | TRAF3IP2 | 0.159284482 | 0.000314953 |
| hsa-miR-150-5p | PLXDC1 | 0.105082551 | 0.096649154 |
| hsa-miR-150-5p | TXK | 0.177538099 | 0.000621514 |
| hsa-miR-150-5p | DSN1 | 0.094761585 | 0.000706213 |
| hsa-miR-150-5p | FAM89A | 0.103520286 | 0.00204297 |
| hsa-miR-150-5p | MASTL | -0.263773793 | 0.000130352 |
| hsa-miR-150-5p | ZNF514 | 0.167730137 | 0.0000842 |
| hsa-miR-150-5p | BBS5 | 0.079411723 | 0.01157737 |
| hsa-miR-150-5p | ASB16 | -0.328815501 | 0.00000129 |
| hsa-miR-150-5p | PPP2CB | 0.168031959 | 0.00000142 |
| hsa-miR-150-5p | POLR3A | -0.237993989 | 0.00000124 |
| hsa-miR-150-5p | HMGB1 | 0.115735705 | 0.008664606 |
| hsa-miR-150-5p | GOSR1 | 0.067689681 | 0.007953998 |
| hsa-miR-150-5p | CEP72 | 0.196155465 | 6.57E-10 |
| hsa-miR-150-5p | BTN3A2 | 0.192690326 | 0.000219053 |
| hsa-miR-150-5p | MYH9 | 0.142721246 | 0.047813391 |
| hsa-miR-150-5p | MMAB | -0.118616898 | 0.009622616 |
| hsa-miR-150-5p | PIGM | 0.199391299 | 0.000000138 |
| hsa-miR-150-5p | WDR53 | 0.126867136 | 0.0000219 |
| hsa-miR-150-5p | SPIB | 0.21340654 | 0.0000432 |
| hsa-miR-150-5p | ATCAY | -0.530383239 | 0.0000124 |
| hsa-miR-150-5p | PGBD4 | -0.081540392 | 0.035863339 |
| hsa-miR-150-5p | ZNF682 | 0.132694859 | 0.020747559 |
| hsa-miR-150-5p | TTC4 | -0.33131728 | 1.75E-08 |
| hsa-miR-150-5p | TIAL1 | 0.050730303 | 0.004623431 |
| hsa-miR-150-5p | TRIM72 | -0.265252831 | 0.00000625 |
| hsa-miR-150-5p | MUT | 0.132018654 | 0.0000205 |
| hsa-miR-150-5p | PPP2R3A | -0.199986854 | 0.003315277 |
| hsa-miR-150-5p | WDR12 | 0.103822018 | 0.001374281 |
| hsa-miR-150-5p | SIGLEC9 | -0.272072952 | 1.26E-11 |
| hsa-miR-150-5p | TRIP11 | 0.151923502 | 0.000000915 |
| hsa-miR-150-5p | RBM3 | 0.243287354 | 0.0000219 |
| hsa-miR-150-5p | KCNK3 | -0.499491174 | 7.28E-11 |
| hsa-miR-150-5p | GK5 | 0.257414011 | 0.000000121 |
| hsa-miR-150-5p | NFYA | 0.166253979 | 0.030511816 |
| hsa-miR-150-5p | PNRC1 | 0.156622365 | 0.000000552 |
| hsa-miR-150-5p | REL | 0.087190436 | 0.00853951 |
| hsa-miR-150-5p | PNPLA3 | -0.134861873 | 0.023758117 |
| hsa-miR-150-5p | RAB13 | -0.095551415 | 0.039876155 |
| hsa-miR-150-5p | POLQ | -0.184723578 | 0.002229182 |
| hsa-miR-150-5p | CBX5 | 0.11161919 | 0.003657114 |
| hsa-miR-150-5p | SUGT1 | 0.113393162 | 0.026622911 |
| hsa-miR-150-5p | SENP8 | 0.143415805 | 0.005199726 |
| hsa-miR-150-5p | KLHL7 | 0.084803108 | 0.010980041 |
| hsa-miR-150-5p | TTC31 | -0.283173136 | 7.87E-09 |
| hsa-miR-150-5p | YPEL1 | 0.152599392 | 0.00000958 |
| hsa-miR-150-5p | CALCOCO2 | 0.087228224 | 0.001298994 |
| hsa-miR-150-5p | TBC1D16 | -0.242833661 | 0.00000913 |
| hsa-miR-150-5p | AMD1 | 0.101249648 | 0.004077092 |
| hsa-miR-150-5p | ANKFY1 | 0.096822568 | 0.00129211 |
| hsa-miR-150-5p | C8orf46 | 0.159441322 | 0.006574711 |
| hsa-miR-150-5p | ZNF207 | 0.170767178 | 0.000000032 |
| hsa-miR-150-5p | ULK2 | 0.129837536 | 0.000205967 |
| hsa-miR-150-5p | RABAC1 | 0.114753991 | 0.00000418 |
| hsa-miR-150-5p | ZNF573 | 0.279084869 | 5.31E-11 |
| hsa-miR-150-5p | C16orf58 | 0.172366202 | 6.37E-12 |
| hsa-miR-150-5p | SPAG16 | 0.07088185 | 0.02793341 |
| hsa-miR-150-5p | EZH2 | 0.203875792 | 0.000000198 |
| hsa-miR-150-5p | WWC2 | 0.099786191 | 0.037561533 |
| hsa-miR-150-5p | TMEM134 | 0.064397537 | 0.019464108 |
| hsa-miR-150-5p | SEC14L4 | -0.330236437 | 0.00000252 |
| hsa-miR-150-5p | RBL1 | 0.212125958 | 0.000511349 |
| hsa-miR-150-5p | PPIE | 0.129341176 | 0.0000926 |
| hsa-miR-150-5p | TRIM35 | -0.173713909 | 0.08737811 |
| hsa-miR-150-5p | LEAP2 | 0.217151582 | 7.02E-09 |
| hsa-miR-150-5p | ZNF551 | 0.264187579 | 2.88E-09 |
| hsa-miR-150-5p | LTBP2 | -0.362175352 | 0.000000032 |
| hsa-miR-150-5p | PSMC1 | 0.119407451 | 0.0000219 |
| hsa-miR-150-5p | FKBP9 | -0.181640136 | 0.005877258 |
| hsa-miR-150-5p | TTYH3 | -0.163079899 | 0.000269942 |
| hsa-miR-150-5p | TNS4 | 0.107398361 | 0.004643523 |
| hsa-miR-150-5p | SYNJ2BP | 0.171271169 | 4.07E-08 |
| hsa-miR-150-5p | SLFN13 | 0.203706545 | 0.0000443 |
| hsa-miR-150-5p | SLC33A1 | 0.179687932 | 0.00000263 |
| hsa-miR-150-5p | SHE | -0.345745155 | 0.0000128 |
| hsa-miR-150-5p | SCO1 | 0.131915068 | 0.00000669 |
| hsa-miR-150-5p | RABIF | 0.094172967 | 0.0000945 |
| hsa-miR-150-5p | PLEKHA2 | 0.080166192 | 0.076224033 |
| hsa-miR-150-5p | NR2F2 | -0.244568291 | 0.003431636 |
| hsa-miR-150-5p | MON1B | -0.360049173 | 0.00000059 |
| hsa-miR-150-5p | C21orf33 | 0.190536946 | 9.1E-09 |
| hsa-miR-150-5p | EPHB2 | -0.140842446 | 0.009975327 |
| hsa-miR-150-5p | DHTKD1 | -0.092880485 | 0.04481342 |
| hsa-miR-150-5p | CNNM2 | -0.153239624 | 0.001304816 |
| hsa-miR-150-5p | CEP135 | 0.116161017 | 0.000000464 |
| hsa-miR-150-5p | BCL11B | 0.244805776 | 1.06E-08 |
| hsa-miR-150-5p | MLN | -0.370886878 | 9.12E-09 |
| hsa-miR-150-5p | TRPV2 | 0.141291832 | 0.0000147 |
| hsa-miR-150-5p | RNF157 | 0.147280189 | 0.004499527 |
| hsa-miR-150-5p | NPHP1 | -0.523896034 | 0.00000755 |
| hsa-miR-150-5p | DEGS1 | 0.080184336 | 0.015382298 |
| hsa-miR-150-5p | GNE | 0.183366751 | 8.15E-09 |
| hsa-miR-150-5p | ALDOA | -0.315816117 | 6.64E-08 |
| hsa-miR-150-5p | SLC25A37 | -0.198133152 | 0.001809856 |
| hsa-miR-150-5p | CSNK1E | 0.262230798 | 6.21E-12 |
| hsa-miR-150-5p | LRRC27 | 0.17597901 | 0.001785336 |
| hsa-miR-150-5p | NPHS1 | -0.13851985 | 0.025597628 |
| hsa-miR-150-5p | EMP2 | -0.170540389 | 0.020747559 |
| hsa-miR-150-5p | C15orf40 | 0.128549383 | 0.000000341 |
| hsa-miR-150-5p | TM4SF5 | -0.479026546 | 6.05E-08 |
| hsa-miR-150-5p | PRPF38A | 0.184179483 | 1.06E-10 |
| hsa-miR-150-5p | PARD6G | -0.168068241 | 0.003426315 |
| hsa-miR-150-5p | ZNF347 | 0.273488272 | 1.47E-08 |
| hsa-miR-150-5p | PDCD4 | 0.13332894 | 7.74E-09 |
| hsa-miR-150-5p | XPOT | 0.222088965 | 0.000000521 |
| hsa-miR-150-5p | SLC1A5 | -0.27652987 | 0.000000346 |
| hsa-miR-150-5p | MANEAL | 0.257302768 | 0.00000154 |
| hsa-miR-150-5p | HIF1AN | -0.193510105 | 0.000130352 |
| hsa-miR-150-5p | RPL24 | 0.22331795 | 6.39E-14 |
| hsa-miR-150-5p | SNX2 | 0.124386995 | 0.0000411 |
| hsa-miR-150-5p | MCTS1 | 0.179668535 | 3.31E-11 |
| hsa-miR-150-5p | RUNDC1 | 0.127287752 | 0.002878985 |
| hsa-miR-150-5p | CD96 | 0.255326991 | 6.61E-09 |
| hsa-miR-150-5p | THAP1 | 0.138223441 | 0.0000106 |
| hsa-miR-150-5p | ZNF7 | 0.196120976 | 7.53E-12 |
| hsa-miR-150-5p | PCP4L1 | -0.200560467 | 0.006723606 |
| hsa-miR-150-5p | MICA | 0.126058872 | 0.002452109 |
| hsa-miR-150-5p | OCIAD1 | 0.20497048 | 8.77E-08 |
| hsa-miR-150-5p | TLR7 | 0.246043777 | 0.0000242 |
| hsa-miR-150-5p | INTS7 | 0.216479048 | 1.78E-08 |
| hsa-miR-150-5p | GPR137B | 0.183181304 | 0.00212401 |
| hsa-miR-150-5p | DCTN5 | 0.173719536 | 0.0000139 |
| hsa-miR-150-5p | MSH3 | 0.19043124 | 2.81E-09 |
| hsa-miR-150-5p | C12orf49 | 0.235591636 | 8.75E-11 |
| hsa-miR-150-5p | C11orf1 | 0.088751563 | 0.008397307 |
| hsa-miR-150-5p | ZNF426 | 0.183474693 | 9.12E-09 |
| hsa-miR-150-5p | RTN2 | -0.179201391 | 0.00000442 |
| hsa-miR-150-5p | HS3ST1 | 0.440652281 | 3.14E-09 |
| hsa-miR-150-5p | VPS53 | 0.123528669 | 0.074851909 |
| hsa-miR-150-5p | USP15 | -0.058292057 | 0.099777655 |
| hsa-miR-150-5p | UBE2V2 | 0.174448687 | 0.00000994 |
| hsa-miR-150-5p | TIMM10 | 0.080661996 | 0.037561533 |
| hsa-miR-150-5p | SERINC1 | 0.11400174 | 0.008432307 |
| hsa-miR-150-5p | RABGAP1L | 0.148681548 | 0.002525222 |
| hsa-miR-150-5p | NSUN4 | -0.516531183 | 1.04E-08 |
| hsa-miR-150-5p | ISG20L2 | 0.067798647 | 0.013133257 |
| hsa-miR-150-5p | DDI2 | 0.146486426 | 0.000142051 |
| hsa-miR-150-5p | CRISPLD2 | -0.198335022 | 0.015139022 |
| hsa-miR-150-5p | ZBTB25 | 0.242431692 | 6.57E-10 |
| hsa-miR-150-5p | ZNF665 | -0.33521747 | 0.000000392 |
| hsa-miR-150-5p | GTF2H3 | 0.234949229 | 2.72E-08 |
| hsa-miR-150-5p | IRAK4 | 0.078640258 | 0.017334472 |
| hsa-miR-150-5p | ZNF578 | 0.18269796 | 0.0000133 |
| hsa-miR-150-5p | FOXRED2 | -0.139303488 | 0.003463714 |
| hsa-miR-150-5p | NOL9 | 0.199067018 | 1.06E-10 |
| hsa-miR-150-5p | RFK | 0.198187712 | 0.000021 |
| hsa-miR-150-5p | METTL8 | 0.297996339 | 2.04E-09 |
| hsa-miR-150-5p | APEX2 | -0.05497731 | 0.010137449 |
| hsa-miR-150-5p | PARD3 | -0.277408034 | 3.25E-10 |
| hsa-miR-150-5p | RBM41 | 0.157278657 | 9.39E-09 |
| hsa-miR-150-5p | RNF34 | 0.119568699 | 0.0000192 |
| hsa-miR-150-5p | PRIM1 | 0.050148595 | 0.03702842 |
| hsa-miR-150-5p | C14orf119 | 0.12335512 | 0.0000863 |
| hsa-miR-150-5p | MAN2B2 | 0.170925625 | 0.0000101 |
| hsa-miR-150-5p | GNB5 | 0.266828095 | 1.47E-08 |
| hsa-miR-150-5p | UBOX5 | 0.130907354 | 0.00873731 |
| hsa-miR-150-5p | ZMAT3 | 0.214692421 | 0.005199726 |
| hsa-miR-150-5p | TMEM127 | 0.059567528 | 0.074851909 |
| hsa-miR-150-5p | SLC6A4 | -0.139299848 | 0.006664531 |
| hsa-miR-150-5p | SH3BP5 | 0.216099547 | 7.28E-11 |
| hsa-miR-150-5p | RPL14 | 0.280203507 | 9.58E-12 |
| hsa-miR-150-5p | PLAA | 0.062567552 | 0.028721893 |
| hsa-miR-150-5p | MRPL37 | 0.137668766 | 0.000000168 |
| hsa-miR-150-5p | GPRIN3 | 0.21581014 | 0.000309583 |
| hsa-miR-150-5p | ETV3 | -0.215231113 | 0.00000175 |
| hsa-miR-150-5p | DNAL1 | 0.108302573 | 0.012937758 |
| hsa-miR-150-5p | DNAJB4 | 0.092053364 | 0.059244985 |
| hsa-miR-150-5p | LDHD | -0.42562754 | 4.23E-09 |
| hsa-miR-150-5p | ATP1B3 | 0.196167069 | 3.34E-09 |
| hsa-miR-150-5p | ZSCAN2 | -0.104575855 | 0.021763344 |
| hsa-miR-150-5p | ENTPD4 | 0.131379089 | 0.0000151 |
| hsa-miR-150-5p | STAC2 | -0.172763688 | 0.002839666 |
| hsa-miR-150-5p | MTMR9 | 0.162197742 | 0.000000208 |
| hsa-miR-150-5p | RAI1 | -0.436196272 | 0.00000357 |
| hsa-miR-150-5p | CDIPT | -0.221850709 | 0.00000275 |
| hsa-miR-150-5p | LYRM7 | 0.234417407 | 1.51E-11 |
| hsa-miR-150-5p | FAHD1 | -0.063888341 | 0.02688704 |
| hsa-miR-150-5p | RHOH | 0.296362206 | 7.53E-12 |
| hsa-miR-150-5p | TRUB2 | 0.248744098 | 6.39E-14 |
| hsa-miR-150-5p | RAB21 | 0.064456868 | 0.021988613 |
| hsa-miR-150-5p | AMOTL2 | -0.154233117 | 0.015202979 |
| hsa-miR-150-5p | PIAS2 | 0.129270219 | 0.000205967 |
| hsa-miR-150-5p | GATAD2B | -0.287271199 | 0.00000432 |
| hsa-miR-150-5p | PXMP4 | 0.064222567 | 0.007596261 |
| hsa-miR-150-5p | ACOX1 | -0.094331788 | 0.05627041 |
| hsa-miR-150-5p | CAMK4 | 0.135622153 | 0.003265244 |
| hsa-miR-150-5p | PRKAB1 | 0.154399603 | 0.00000175 |
| hsa-miR-150-5p | RAPGEF6 | 0.20934721 | 0.0000643 |
| hsa-miR-150-5p | CARHSP1 | -0.142611732 | 0.000000211 |
| hsa-miR-150-5p | TEAD1 | -0.553272628 | 5.12E-09 |
| hsa-miR-150-5p | XPNPEP3 | -0.358123608 | 7.44E-08 |
| hsa-miR-150-5p | ZHX3 | 0.225409932 | 7.09E-09 |
| hsa-miR-150-5p | TMEM174 | -0.100615725 | 0.01200769 |
| hsa-miR-150-5p | NEK8 | -0.333294998 | 0.000000393 |
| hsa-miR-150-5p | ATAD2B | 0.149958877 | 0.0000124 |
| hsa-miR-150-5p | CREB1 | 0.097278968 | 0.000699572 |
| hsa-miR-150-5p | STAT5B | -0.137761404 | 0.001194551 |
| hsa-miR-150-5p | STAT1 | -0.088953493 | 0.029065748 |
| hsa-miR-150-5p | POLD3 | 0.088297515 | 0.017275972 |
| hsa-miR-150-5p | PRKCA | 0.217905604 | 4.23E-09 |
| hsa-miR-150-5p | ARRB2 | -0.115617426 | 0.006664531 |
| hsa-miR-150-5p | ZNF350 | 0.098018907 | 0.012197605 |
| hsa-miR-150-5p | SLC2A1 | 0.136965488 | 0.00000412 |
| hsa-miR-150-5p | DDOST | 0.137118187 | 0.00000784 |
| hsa-miR-150-5p | KCTD20 | 0.070865018 | 0.030665268 |
| hsa-miR-150-5p | RAB3IP | 0.120288304 | 0.00000139 |
| hsa-miR-150-5p | STK11 | -0.127027533 | 0.00045728 |
| hsa-miR-150-5p | CDC14B | 0.117349957 | 0.05433882 |
| hsa-miR-150-5p | GJC1 | -0.405379241 | 1.99E-08 |
| hsa-miR-150-5p | GMEB1 | 0.102044608 | 0.000108176 |
| hsa-miR-150-5p | GPBP1 | 0.159013131 | 0.0000012 |
| hsa-miR-150-5p | KIF3A | 0.172901037 | 3.74E-08 |
| hsa-miR-150-5p | KPNA6 | -0.182783056 | 0.000000591 |
| hsa-miR-150-5p | MSRB2 | 0.082915256 | 0.094822859 |
| hsa-miR-150-5p | RABL3 | -0.114244782 | 0.002158603 |
| hsa-miR-150-5p | THAP6 | 0.130113783 | 0.000914547 |
| hsa-miR-150-5p | TLR10 | 0.123605487 | 0.012609526 |
| hsa-miR-150-5p | TOR1AIP1 | 0.057838547 | 0.069453941 |
| hsa-miR-150-5p | TPMT | 0.167711822 | 0.000413891 |
| hsa-miR-150-5p | TRIM65 | -0.089717048 | 0.012512365 |
| hsa-miR-150-5p | ZFP14 | 0.113927547 | 0.006664531 |
| hsa-miR-150-5p | ZNF70 | 0.239242418 | 0.00000105 |
| hsa-miR-150-5p | LRRC58 | 0.149406638 | 0.004335635 |
| hsa-miR-150-5p | MRPS10 | 0.163051399 | 2.95E-10 |
| hsa-miR-150-5p | SP2 | 0.116089633 | 0.003214625 |
| hsa-miR-150-5p | ZNF786 | 0.276767658 | 3.67E-10 |
| hsa-miR-627 | SKI | -0.253734027 | 0.048819464 |
| hsa-miR-627 | C9orf66 | 0.201279883 | 0.034858404 |
| hsa-miR-627 | FOS | 0.264921564 | 0.096492105 |
| hsa-miR-627 | SGTB | 0.103964275 | 0.055848864 |
| hsa-miR-627 | NLRP9 | 0.336459077 | 0.002774901 |
| hsa-miR-627 | CD55 | 0.208363401 | 0.007149839 |
| hsa-miR-627 | PTPRJ | 0.287661823 | 0.0000498 |
| hsa-miR-627 | PRRG4 | 0.205689237 | 0.007149839 |
| hsa-miR-627 | OGFRL1 | 0.174200599 | 0.019424987 |
| hsa-miR-627 | NINJ1 | 0.265918234 | 0.002590005 |
| hsa-miR-627 | RAB11FIP1 | 0.178204867 | 0.02953054 |
| hsa-miR-627 | TREM1 | 0.357694821 | 0.002590005 |
| hsa-miR-627 | TLR8 | 0.207970575 | 0.020102827 |
| hsa-miR-627 | NOTCH1 | 0.185922428 | 0.004921411 |
| hsa-miR-627 | USP15 | 0.132009669 | 0.012948225 |
| hsa-miR-627 | KREMEN1 | 0.384914298 | 0.007149839 |
| hsa-miR-627 | LRRC4 | 0.308790198 | 0.005131236 |
| hsa-miR-664b-3p | PARD6B | 0.290646714 | 0.00062609 |
| hsa-miR-664b-3p | LBR | 0.13918179 | 0.01712606 |
| hsa-miR-664b-3p | GLO1 | 0.234486754 | 0.001937448 |
| hsa-miR-664b-3p | STARD7 | 0.176141207 | 0.013165476 |
| hsa-miR-664b-3p | DEK | 0.1731007 | 0.001638376 |
| hsa-miR-664b-3p | ANKRD17 | 0.085979467 | 0.058220904 |
| hsa-miR-664b-3p | PGLS | 0.07513166 | 0.087077196 |
| hsa-miR-664b-3p | TGOLN2 | 0.131811618 | 0.012380267 |
| hsa-miR-664b-3p | RNF11 | -0.180095559 | 0.002649226 |
| hsa-miR-664b-3p | ARL6IP6 | 0.126482947 | 0.033137927 |
| hsa-miR-664b-3p | RBM12B | 0.159227566 | 0.007965322 |
| hsa-miR-664b-3p | RAN | 0.191774506 | 0.000964199 |
| hsa-miR-664b-3p | ZNF507 | 0.159549928 | 0.060117654 |
| hsa-miR-664b-3p | MAGEL2 | -0.421818973 | 0.000206884 |
| hsa-miR-664b-3p | PPIL3 | 0.090826398 | 0.076356115 |
| hsa-miR-664b-3p | ZNF331 | 0.092220751 | 0.076384103 |
| hsa-miR-664b-3p | RSRC1 | -0.199437469 | 0.000964199 |
| hsa-miR-664b-3p | PHIP | 0.103952752 | 0.084857102 |
| hsa-miR-664b-3p | RCC2 | 0.11110557 | 0.077481454 |
| hsa-miR-664b-3p | FANCF | 0.210646661 | 0.001618473 |
| hsa-miR-664b-3p | SETD5 | -0.543326778 | 0.001856997 |
| hsa-miR-664b-3p | PAK4 | -0.319033188 | 0.001937448 |
| hsa-miR-664b-3p | MDC1 | 0.252025398 | 0.0000485 |
| hsa-miR-664b-3p | CHD4 | 0.092795978 | 0.023568605 |
| hsa-miR-664b-3p | ZBTB7A | -0.125351829 | 0.013165476 |
| hsa-miR-664b-3p | FAM84B | 0.342054451 | 0.01712606 |
| hsa-miR-664b-3p | ZNF616 | 0.104194221 | 0.04750293 |
| hsa-miR-664b-3p | TNFSF8 | -0.127296545 | 0.043966575 |
| hsa-miR-664b-3p | ZCCHC2 | 0.237984611 | 0.001638376 |
| hsa-miR-664b-3p | SLC41A1 | 0.240580882 | 0.000318717 |
| hsa-miR-664b-3p | PNRC1 | 0.195667907 | 0.0000769 |
| hsa-miR-664b-3p | PKIA | 0.128153118 | 0.021485109 |
| hsa-miR-664b-3p | LZIC | 0.065067259 | 0.064809485 |
| hsa-miR-664b-3p | CDKN2AIP | 0.182842814 | 0.000093 |
| hsa-miR-664b-3p | TBCA | 0.224879357 | 0.0000485 |
| hsa-miR-664b-3p | ZNF514 | 0.145237587 | 0.033425117 |
| hsa-miR-664b-3p | SHMT1 | -0.206197294 | 0.04750293 |
| hsa-miR-664b-3p | NAV2 | -0.195974083 | 0.046328233 |
| hsa-miR-664b-3p | MAPK14 | 0.094155222 | 0.083426261 |
| hsa-miR-664b-3p | CREB1 | 0.12271855 | 0.004803342 |
| hsa-miR-664b-3p | GTF2H5 | 0.148296283 | 0.00200145 |
| hsa-miR-664b-3p | SLC6A5 | -0.348260524 | 0.003904512 |
| hsa-miR-664b-3p | ZNF185 | -0.133966356 | 0.064039291 |
| hsa-miR-664b-3p | CXorf56 | 0.139768745 | 0.060117654 |
| hsa-miR-664b-3p | COX6A1 | 0.136858013 | 0.0000772 |
| hsa-miR-664b-3p | TMED4 | -0.262713985 | 0.05062904 |
| hsa-miR-664b-3p | ITGBL1 | -0.443071385 | 0.004508627 |
| hsa-miR-664b-3p | RBM41 | 0.080092094 | 0.073756386 |
| hsa-miR-664b-3p | ZNF264 | 0.289492983 | 0.000000831 |
| hsa-miR-664b-3p | GPBP1 | 0.136170886 | 0.008490218 |
| hsa-miR-664b-3p | SLC5A12 | -0.490352101 | 0.005258516 |
| hsa-miR-664b-3p | DDAH1 | -0.305288132 | 0.001618473 |
| hsa-miR-664b-3p | RNASEL | 0.115441629 | 0.034085721 |
| hsa-miR-664b-3p | CYFIP2 | 0.116932439 | 0.004803342 |
| hsa-miR-664b-3p | DMXL1 | 0.246004874 | 0.000206884 |
| hsa-miR-664b-3p | REEP5 | 0.139872459 | 0.002649226 |
| hsa-miR-664b-3p | UQCRFS1 | 0.151320426 | 0.001638376 |

**Supplementary Table S4**. The AD related KEGG pathways and the most relevant supportive literature.

| **KEGG Pathway** | **AD-related supported literature** |
| --- | --- |
| Hippo signaling pathway (human) | DOI: 10.1002/jnr.24551 |
| Adipocytokine signaling pathway (human) | DOI: 10.1007/s11011-014-9501-z |
| Fatty acid degradation (human) | DOI: 10.1016/j.neurobiolaging.2019.03.001 |
| Base excision repair (human) | DOI: 10.1016/S0006-8993(99)02335-5 |
| Tight junction (human) | DOI: 10.1093/brain/awz011 |
| Axon guidance (human) | DOI: 10.5607/en.2019.28.3.311 |
| Prolactin signaling pathway (human) | DOI: 10.1159/000517798 |
| Chemokine signaling pathway (human) | DOI: 10.1177/1533317513518651 |
| Thyroid hormone signaling pathway (human) | DOI: 10.3389/fendo.2014.00062 |
| Dopaminergic synapse (human) | DOI: 10.3389/fnagi.2014.00252 |
| Herpes simplex virus 1 infection (human) | DOI: 10.3389/fnagi.2018.00048 |
| Circadian rhythm (human) | DOI: 10.3390/medsci6030052 |
| Huntington disease (human) | DOI: 10.4161/cl.21602 |
| TNF signaling pathway (human) | DOI: 10.1023/A:1022337519035 |
| NOD-like receptor signaling pathway (human) | DOI: 10.1002/jnr.24004 |
| Pathways of neurodegeneration - multiple diseases (human) | DOI: 10.1038/nm1067 |
| Human cytomegalovirus infection (Human) | DOI: 10.1093/infdis/jit210 |
| Thermogenesis (Human) | DOI: 10.1016/j.molmet.2019.01.008 |
| PPAR signaling pathway (human) | DOI: 10.2174/157015911798376325 |
| Sphingolipid signaling pathway (human) | DOI: 10.1007/s12017-010-8121-y |
| Nucleotide excision repair (human) | DOI: 10.1016/j.neulet.2018.02.043 |
| Prion disease (human) | DOI: https://doi.org/10.1097/NEN.0000000000000228 |
| Oxidative phosphorylation (human) | DOI: https://doi.org/10.1385/NMM:5:2:147 |

**Supplementary Table S5**. The regulation relation among these miRNAs, TFs and gene targets based on TransmiR v2 and TRRUST v2 databases.

| **TFs** | **Targets** | **Regulation** | **Evidence** | **Tissue** | **Sourse** |
| --- | --- | --- | --- | --- | --- |
| CREB1 | hsa-mir-3651 | Regulation | level 1 | Blood | TransmiR v2 |
| CREB1 | hsa-mir-3651 | Regulation | level 2 | Liver | TransmiR v2 |
| CREB1 | hsa-mir-3651 | Regulation | level 1 | Liver | TransmiR v2 |
| CREB1 | hsa-mir-3651 | Regulation | level 2 | Lung | TransmiR v2 |
| CREB1 | hsa-mir-3651 | Regulation | level 1 | Pluripotent stem cell | TransmiR v2 |
| CREB1 | hsa-mir-3651 | Regulation | level 2 | Prostate | TransmiR v2 |
| CREB1 | hsa-mir-3651 | Regulation | level 1 | Prostate | TransmiR v2 |
| CREB1 | hsa-mir-627 | Regulation | level 2 | Blood | TransmiR v2 |
| CREB1 | hsa-mir-627 | Regulation | level 2 | Liver | TransmiR v2 |
| CREB1 | hsa-mir-664b | Regulation | level 2 | Prostate | TransmiR v2 |
| EP300 | hsa-mir-150 | Regulation(feedback) | level 1 | Blood | TransmiR v2 |
| EP300 | hsa-mir-150 | Activation(feedback) | literature(26644403) | None | TransmiR v2 |
| EP300 | hsa-mir-3651 | Regulation | level 2 | Blood | TransmiR v2 |
| EP300 | hsa-mir-3651 | Regulation | level 1 | Blood | TransmiR v2 |
| EP300 | hsa-mir-3651 | Regulation | level 1 | Kidney | TransmiR v2 |
| EP300 | hsa-mir-3651 | Regulation | level 2 | Neural | TransmiR v2 |
| EP300 | hsa-mir-3651 | Regulation | level 2 | Pluripotent stem cell | TransmiR v2 |
| EP300 | hsa-mir-627 | Regulation | level 1 | Blood | TransmiR v2 |
| EZH2 | hsa-mir-627 | Regulation | level 1 | Prostate | TransmiR v2 |
| EZH2 | hsa-mir-664b | Regulation | level 1 | Prostate | TransmiR v2 |
| FOS | hsa-mir-3651 | Regulation | level 2 | Blood | TransmiR v2 |
| FOS | hsa-mir-3651 | Regulation | level 2 | Breast | TransmiR v2 |
| FOS | hsa-mir-3651 | Regulation | level 2 | Uterus | TransmiR v2 |
| MYB | hsa-mir-150 | Regulation(feedback) | level 1 | Blood | TransmiR v2 |
| MYB | hsa-mir-3651 | Regulation | level 1 | Blood | TransmiR v2 |
| NFYA | hsa-mir-3651 | Regulation | level 2 | Blood | TransmiR v2 |
| NFYA | hsa-mir-3651 | Regulation | level 1 | Digestive tract | TransmiR v2 |
| NFYA | hsa-mir-3651 | Regulation | level 2 | Uterus | TransmiR v2 |
| NFYA | hsa-mir-664b | Regulation | level 1 | Blood | TransmiR v2 |
| NOTCH1 | hsa-mir-150 | Regulation | level 1 | Blood | TransmiR v2 |
| NOTCH1 | hsa-mir-3651 | Regulation | level 1 | Blood | TransmiR v2 |
| NOTCH1 | hsa-mir-627 | Regulation | level 1 | Blood | TransmiR v2 |
| NOTCH1 | hsa-mir-664b | Regulation | level 1 | Blood | TransmiR v2 |
| NR2F2 | hsa-mir-3651 | Regulation | level 2 | Liver | TransmiR v2 |
| SP2 | hsa-mir-627 | Regulation | level 2 | Kidney | TransmiR v2 |
| SP2 | hsa-mir-664b | Regulation | level 2 | Kidney | TransmiR v2 |
| STAT1 | hsa-mir-3651 | Regulation | level 2 | Blood | TransmiR v2 |
| STAT1 | hsa-mir-3651 | Regulation | level 1 | Blood | TransmiR v2 |
| STAT1 | hsa-mir-627 | Regulation | level 2 | Blood | TransmiR v2 |
| STAT1 | hsa-mir-627 | Regulation | level 1 | Blood | TransmiR v2 |
| STAT1 | hsa-mir-664b | Regulation | level 1 | Blood | TransmiR v2 |
| TP53 | hsa-mir-150 | Repression(feedback) | literature(25757558) | None | TransmiR v2 |
| TP53 | hsa-mir-3651 | Regulation | level 1 | Breast | TransmiR v2 |
| TP53 | hsa-mir-3651 | Regulation | level 2 | Digestive tract | TransmiR v2 |
| CREB1 | ETV3 | Activation | 14754893 |  | TRRUST v2 |
| CREB1 | FOS | Unknown | 12432566 |  | TRRUST v2 |
| EP300 | EZH2 | Unknown | 25088689 |  | TRRUST v2 |
| EZH2 | TP53 | Repression | 20132185 |  | TRRUST v2 |
| EZH2 | TP53 | Unknown | 24006445 |  | TRRUST v2 |
| FOS | FOS | Repression | 2498646 |  | TRRUST v2 |
| FOS | TP53 | Unknown | 10348347 |  | TRRUST v2 |
| MYB | MYB | Unknown | 7655015 |  | TRRUST v2 |
| STAT1 | TP53 | Activation | 14602726 |  | TRRUST v2 |
| STAT1 | TP53 | Unknown | 24655378 |  | TRRUST v2 |
| TP53 | EZH2 | Activation | 15208672 |  | TRRUST v2 |
| TP53 | EZH2 | Repression | 15208672;21165554 |  | TRRUST v2 |
| TP53 | STAT1 | Unknown | 12364590 |  | TRRUST v2 |
| TP53 | TP53 | Activation | 22532570 |  | TRRUST v2 |
| TP53 | TP53 | Unknown | 11032029;16990849 |  | TRRUST v2 |
| ZBTB7A | FOS | Repression | 17189472 |  | TRRUST v2 |
